# Gut microbiota as a predictive tool for outcomes in IgA nephropathy

**DOI:** 10.1080/0886022X.2025.2514184

**Published:** 2025-07-09

**Authors:** Yijun Dong, Ge Yan, Yiding Zhang, Yukun Zhou, Jin Shang

**Affiliations:** a Department of Nephrology, the First Affiliated Hospital of Zhengzhou University, Zhengzhou, China; b School of Medicine, Zhengzhou University, Zhengzhou, China; c Laboratory Animal Platform of Academy of Medical Sciences, Zhengzhou University, Zhengzhou, China

**Keywords:** IgA nephropathy, gut microbiota, microbial dysbiosis, Predictive model, treatment response

## Abstract

Immunoglobulin A nephropathy (IgAN) is characterized by the deposition of glycosylation-deficient IgA1 in the glomeruli and has been linked to the gut-kidney axis. This study aimed to determine if baseline differences in gut microbiota could predict therapeutic responses in IgAN patients. We analyzed fecal microbiomes of 55 biopsy-confirmed IgAN patients and followed them for over 6 months. Patients were classified as responders (*n* = 39) or nonresponders (*n* = 16) based on remission status. Fecal microbiomes were profiled using 16S rRNA sequencing, revealing significant microbiota differences. Nonresponders had increased *Proteobacteria* and *Firmicutes*, with notable enrichment of opportunistic bacteria like *Escherichia-Shigella* and *Pseudomonas*. A predictive classifier based on 24 amplicon sequence variants, with *Escherichia-Shigella* and *Pseudomonas* as key contributors, showed high accuracy in identifying nonresponders (AUC 0.9103, *p* < 0.0001). These findings highlight the role of microbial dysbiosis in IgAN progression and treatment response, suggesting that gut microbiota analysis could guide personalized therapy for IgAN. Future studies with larger cohorts are needed to validate these results and explore microbiome-based treatments.

## GRAPHICAL ABSTRACT

**Figure:**
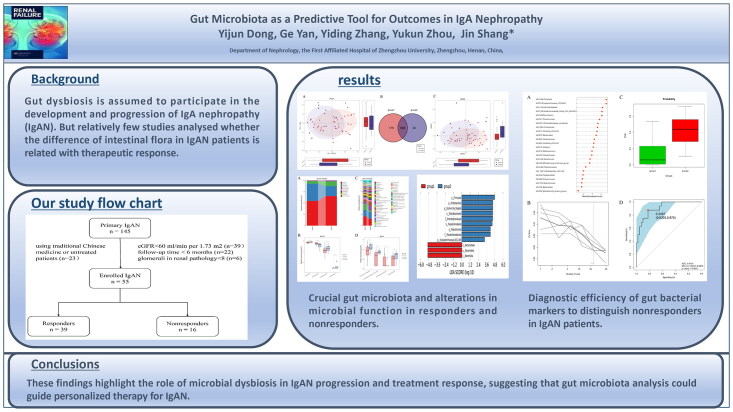

## 1. Introduction

Immunoglobulin A (IgA) nephropathy (IgAN) is the most prevalent primary glomerulonephritis internationally, mainly affecting 20–40 years old people [1]. In particular, 20–40% of patients will progress to end-stage renal disease in about 20 years after diagnosis [2]. Pathologically, it is characterized by the diffuse deposition of immune complexes formed by glycosylation-deficient IgA1 (Gd-IgA1) and its specific antibodies (Gd-IgA1-IgG) in the mesangial region of the glomeruli. Although the causes and mechanisms of Gd-IgA1 production remain unclear, increasing evidence indicates that the plasma cells producing Gd-IgA1 mainly originate from gut-associated lymphoid tissue, forming the basis of the ‘gut-kidney axis’ theory [3–5]. Subsequent studies have revealed significant differences in the composition of gut microbiota and their metabolites between IgAN patients and healthy individuals [6]. Further researches suggest that gut microbiota may play a role in the onset and progression of IgA [7]. Several interventions targeting gut microbiota, such as the use of probiotic [8], fecal microbiota transplantation [9], and modulation of gut immunity, have shown therapeutic potential for IgAN. Among these, oral budesonide controlled-release capsules, designed for targeted delivery to the terminal ileum, have been proven to reduce proteinuria levels and delay renal failure in IgAN patients [10]. Increasing evidence suggests that gut immunity may play a critical role in the onset and progression of IgAN, and gut microbiota may influence disease onset, treatment response, and prognosis in IgAN patients by modulating gut immunity.

Managing IgA nephropathy is particularly challenging. Little is known about the long-term outcomes and risk factors that influence kidney recovery in IgAN patients, especially those patients who have undergone initial aggressive immunosuppressive therapy. Proteinuria severity at diagnosis has consistently been identified as a risk factor for kidney function decline [11]. However, findings from the STOP-IgA nephropath trial and the TESTING trial indicated no significant differences in the effectiveness of immunosuppression relative to baseline proteinuria levels [11]. Additionally, systemic immunosuppressive therapies are associated with considerable toxicity. In the absence of reliable biomarkers, it remains difficult to pinpoint patients with active disease who are at high risk of progression and could benefit most from immunosuppressive treatment. To address this, we analyzed the long-term outcomes of 55 biopsy-confirmed IgA nephropathy patients who were treated with glucocorticoid and/or immunosuppresors. and developed a predictive model based on gut microbiota to assess disease outcomes.

## 2. Materials and methods

### 2.1. Study design and participants

Participant Information: The study followed the PRoBE (Prospective Specimen Collection and Retrospective Blinded Evaluation) design principle [12], which involves prospective specimen collection combined with a retrospective, blinded analysis. This study was approved by the Ethics Committee of the First Affiliated Hospital of Zhengzhou University (Approval No. 2022-KY-0572-001) and conducted in accordance with the principles outlined in the Declaration of Helsinki. All participants provided signed informed consent at the time of enrollment. Patients in our study received standard therapy based on clinical guidelines, including: prednisone alone or in combination with immunosuppressants such as tacrolimus, mycophenolate mofetil, and cyclophosphamide; no patient had received immunosuppressive therapy prior to biopsy, ensuring that baseline gut microbiota composition was not influenced by prior treatment. All patients were followed up for at least 6 months to assess treatment response. Demographic and clinical information was collected through questionnaires and extracted from the hospitals’ electronic medical records.

Inclusion criteria considering the following cases: (1) primary IgAN confirmed by biopsy; (2) an estimated glomerular filtration rate (eGFR) greater than 60 mL/min/1.73 m^2^, to minimize the impact of advanced CKD on gut microbiota composition.

Exclusion criteria considering the following cases: (1) applied immunosuppressive treatment before biopsy, using traditional Chinese medicine or untreated after biopsy; (2) had received probiotics or antibiotics before inclussion less 3 months; (3) had co-morbidities, such as other autoimmune diseases, acute or chronic gastrointestinal diseases, cancer; (4) follow-up time <6 months; (5) the number of glomeruli in renal pathology < 8.

Partial remission and complete remission patients both were defined as responders. Partial remission was defined by 24-h proteinuria (reduced to < 50% of the baseline and maintained > 1.0 g/day) and stable renal function (eGFR reduction < 30% of the baseline). Complete remission was defined by 24-h proteinuria (reduced to < 0.3 g/day) and stable renal function (eGFR reduction < 30% of the baseline) [13]. Hypertension was defined by a systolic blood pressure (SBP) ≥ 130 or a diastolic blood pressure (DBP) ≥ 80 mmHg [14].

### 2.2. Fecal sample collection and bacterial taxon identification

Every enrolled individuals handed in fresh fecal samples in the morning before the initiation of immunosuppressive therapy. Proper amount of fecal samples were immediately stored at −80 °C. The DNA extraction was conducted according to the instruction of previous study [15]. In short, the bacterial genomic DNA was extracted by the E.Z.N.A.Stool DNA Kit (omega, American). DNA concentration (by NanoDrop, Thermo Scientific) and the molecular size (by agarose gel electrophoresis) were measured too. The high-variant v3–v4 region of 16S rRNA gene were amplified, sequenced and analyzed using Miseq platform (Illumina Inc., USA) (2 × 300 cycles run) as previously described [16].

### 2.3. Statistical analysis

Alpha diversity (including ace, chao, Shannon and Simpson index) and β diversity were conducted by the R package as previously described [17]. By a sampling-based amplicon sequence variants (ASVs) Analysis, the Venn diagram was used to show the microbiome space among the group samples. The linear discriminant analysis (LDA) effect size (LEfSe) method (lefse 1.1, https://github.com/SegataLab/lefse) was used to analyze fecal microbial characterization and explain the differences among the different groups. The PICRUSt2 (v2.4.1, https://github.com/picrust/picrust2/wiki) was used to construct KEGG orthology (KO) and the KEGG pathway/module profile on the basis of ASV marker gene sequences. The significance of the discovery ASVs was determined by wilcoxon test (*p* < 0.05). The optimal ASVs set was obtained by using five-fold cross-validation (R 3.4.1, random forest 4.6–12 package). The probability of disease (POD) index was set up to predicted whether samples were from responders or nonresponders. The receiver operating characteristic (ROC) curve (R 3.4.1, pROC package) was generated for the evaluation of the constructed models. Meanwhile, the area under curve (AUC) was determined to to assess diagnostic power of the model.

Statistical analyses were performed using SPSS (ver.26.0) and R software (ver. 3.1.0). Quantitative data with normal distribution was described by the mean and SD, and non-normal distribution data by median with 25th and 75th quartiles. Continuous variables were compared by Wilcoxon rank sum test. A multivariable logistic regression analysis was performed. The odds ratio (OR) and 95% confidence interval (CI) were calculated for each variable, and statistical significance was set at *p* < 0.05.

### 2.4. Ethics approval and consent to participate

The study was approved by the Institutional Review Board of the First Affiliated Hospital of Zhengzhou University (2022-KY-0572-001) and conducted in accordance with the Declaration of Helsinki. Written informed consent was obtained from all study participants.

## 3. Results

### 3.1. Study population and classification of responders and nonresponders

**Figure 1.**
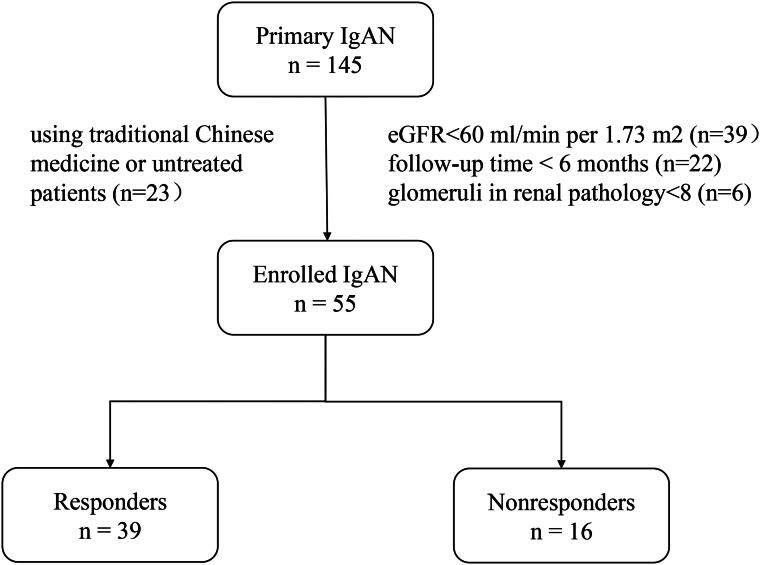
Study design and flow chart.

We retrospectively enrolled 145 patients with primary IgAN at the First Affiliated Hospital of Zhengzhou University between May 2018 and July 2021. Of these, 55 patients who met the inclusion criteria were included in the study. Patients who received steroids or immunosuppressive therapy were followed for at least 6 months. They were classified as responders if they achieved complete or partial remission following treatment. Those who did not meet these criteria were categorized as nonresponders (Figure 1).

### 3.2. Clinical characteristics of responders and nonresponders

Compared to nonresponders, there were no significant differences in sex, BMI, or age among the responders (*p* > 0.05; Table 1). Additionally, there were some differences in serum creatinine, urea nitrogen, and proteinuria between responders and nonresponders, but they did not reach statistical significance (*p* > 0.05; Table 1).

**Table 1.** Clinical parameters of IgAN patients with and without clinical remission.

Parameters	Responders (*n* = 39)	Nonresponders (*n* = 16)	*p* Value
Age, year	39.29 ± 14.17	40.44 ± 9.06	0.721
Male, *n* (%)	17 (43.6%)	11 (68.8%)	0.090
BMI, kg/m^2^	23.83 ± 3.61	25.38 ± 1.65	0.572
Hypertension, *n* (%)	13 (33.3%)	4 (25%)	0.721
Anemia, *n* (%)	18 (46.2%)	4 (25%)	0.750
Albumin, g/L	37.70 (35.90,41.50)	40.80 (39.93,41.63)	0.037
eGFR, ml/min per 1.73 m^2^	88.56 (74.17,105.11)	72.86 (64.18,105.80)	0.120
Serum creatinine, μmol/L	81.20 ± 17.81	95.12 ± 26.65	0.068
Urea nitrogen, mmol/L	5.56 ± 1.69	6.50 ± 1.90	0075
Uric acid, μmol/L	326.36 ± 81.16	333.19 ± 112.37	0.802
Proteinuria, g/day	1.60 (0.88,2.68)	0.90 (0.48,1.65)	0.052
Renal histologic lesion, *n* (%)			
M1	12 (30.8%)	4 (25.0%)	0.754
E1	16 (40.1%)	3 (18.8%)	0.206
S1	31 (79.5%)	10 (62.5%)	0.306
T lesion			
T0	37 (94.9%)	13 (81.3%)	0.141
T1	2 (5.1%)	2 (12.5%)	0.571
T2	0 (0.00%)	1 (6.3%)	0.291
C lesion			
C0	26 (66.7%)	13 (81.3%)	0.344
C1	12 (30.8%)	3 (18.8%)	0.510
C2	1 (2.6%)	0 (0.00%)	1.000
Prednison, *n* (%)	9 (23.1%)	1 (6.3%)	0.250
Immunosuppressants, *n* (%)	6 (15.4%)	4 (25%)	0.453
Prednison combined with immunosuppressants, *n* (%)	10 (25.6%)	1 (6.3%)	0.146

M1: mesangial hyperplasia; E1: endocapillary hyperplasia.

S1: segmental glomerulosclerosis; T: tubular atrophy and/or interstitial fibrosis; T0: tubular atrophy less than 25%; T1: tubular atrophy less than 26% and more than 50%; T2: tubular atrophy more than 50%; C0: no crescents; C1: crescents in less than.

25% of glomeruli; C2: crescents in over 25% of glomeruli.

To further assess whether these clinical parameters independently influenced gut dysbiosis, we conducted a multivariable logistic regression analysis (Table 2). The results showed that none of the examined variables reached statistical significance (*p* > 0.05).

**Table 2.** Multivariable logistic regression analysis for gut dysbiosis prediction.

	*B*	Standard Error	Wald	*p* Value	OR (95% CI)
Age, year	−0.248	0.15	2.739	0.098	0.781(0.582,1.047)
Urea nitrogen, mmol/L	−0.072	0.386	0.035	0.853	0.931(0.437,1.984)
Serum creatinine, μmol/L	0.179	0.233	0.59	0.443	1.196(0.757,1.889)
Uric acid, μmol/L	−0.009	0.009	0.881	0.348	0.991(0.974,1.009)
Albumin, g/L	−0.02	0.099	0.042	0.838	0.98(0.807,1.19)
eGFR, mL/min per 1.73 m^2^	−0.225	0.196	1.311	0.252	0.799(0.544,1.173)
Proteinuria, g/day	−0.684	0.377	3.285	0.07	0.505(0.241,1.057)
Male, *n* (%)	2.142	1.946	1.212	0.271	8.52(0.188,386.526)
Hypertension, *n* (%)	0.29	0.932	0.097	0.755	1.337(0.215,8.297)
Anemia, *n* (%)	0.119	1.221	0.01	0.922	1.127(0.103,12.337)

### 3.3. Characteristic of gut microbiome dysbiosis in nonresponders

Multiple taxa were detected, with each bacterial diversity approaching saturation, as indicated by Shannon-Wiener analysis (Supplemental Figure 1(A)). In responders and nonresponders, no significant differences were observed in the abundance and diversity of gut microbiota in alpha diversity (Supplemental Figure 1(B–F), Supplemental Table 1), this result should be confirmed in larger sample studies due to the relatively small sample size. In contrast, β-diversity revealed a significant distinction in gut microbiota composition between responders and nonresponders, as demonstrated by principal coordinate analysis (PCoA) (Figure 2(A)) and nonmetric multidimensional scaling (NMDS) plots (Figure 2(C)). A total of 773 and 628 amplicon sequence variants (ASVs) were identified in respectively, with 598 shared ASVs (Figure 2(B)).

Figure 2.Gut Microbiome dysbiosis in nonresponders.

**Figure:**
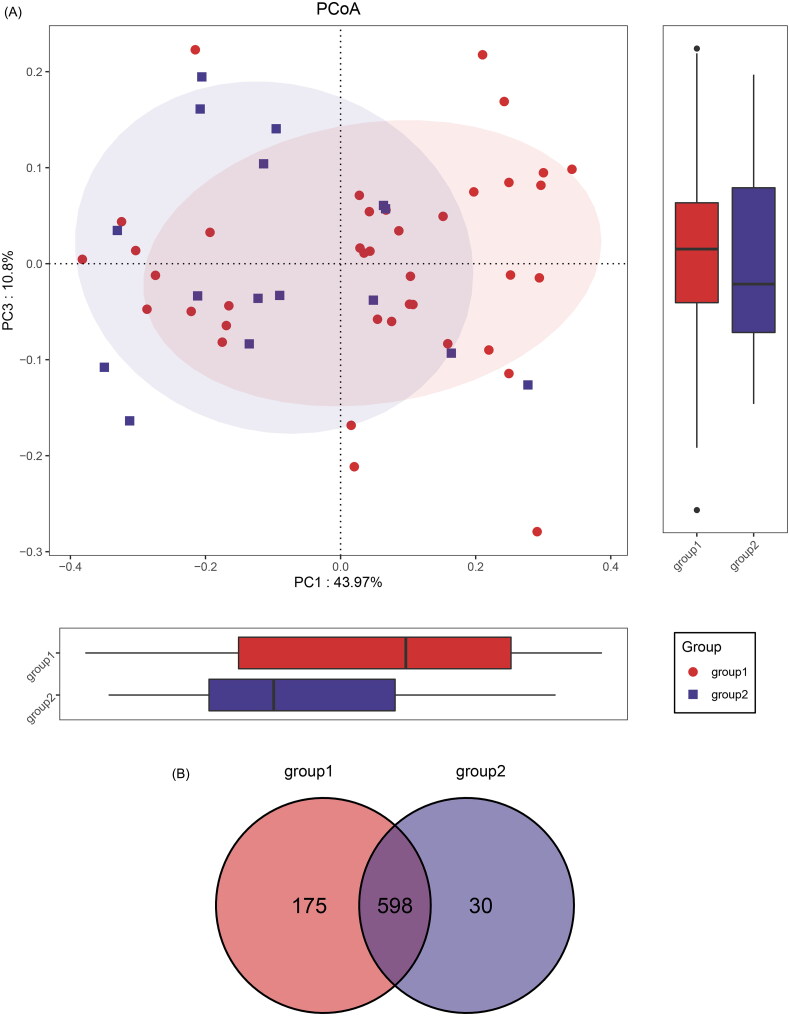

**Figure:**
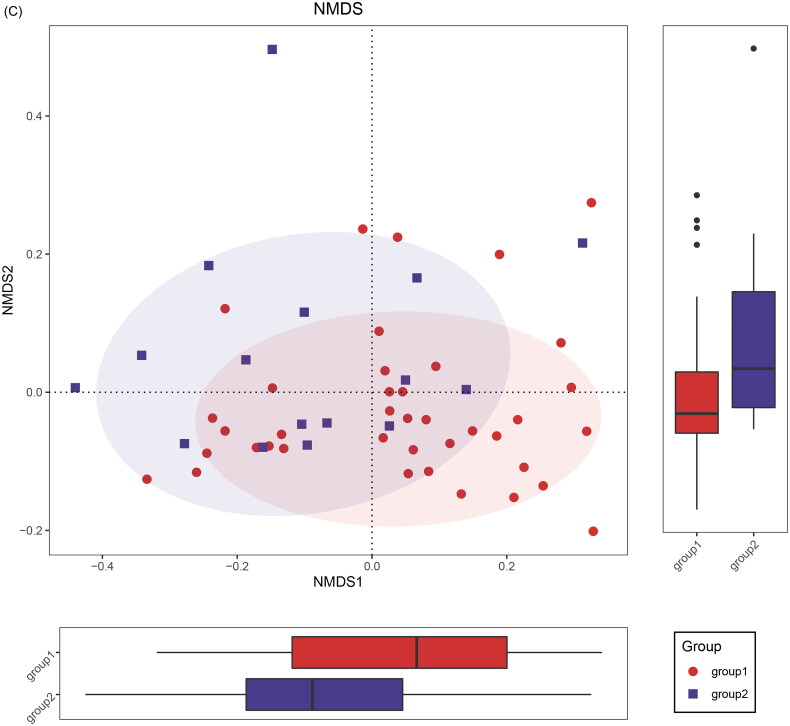

Next, we observed distinct microbiota profiles at various taxonomic levels between responders and nonresponders. At the phylum level, *Proteobacteria* and *Firmicutes* were significantly enriched in nonresponders compared to responders (Figure 3(A,B)). At the genus level, there was an increase in certain opportunistic bacteria, including *Escherichia-Shigella*, *Pseudomonas*, and *Erysipelotrichaceae_UCG_003* (Figure 3(C,D).

Figure 3.Crucial Gut Microbiota and alterations in microbial function in responders and nonresponders.

**Figure:**
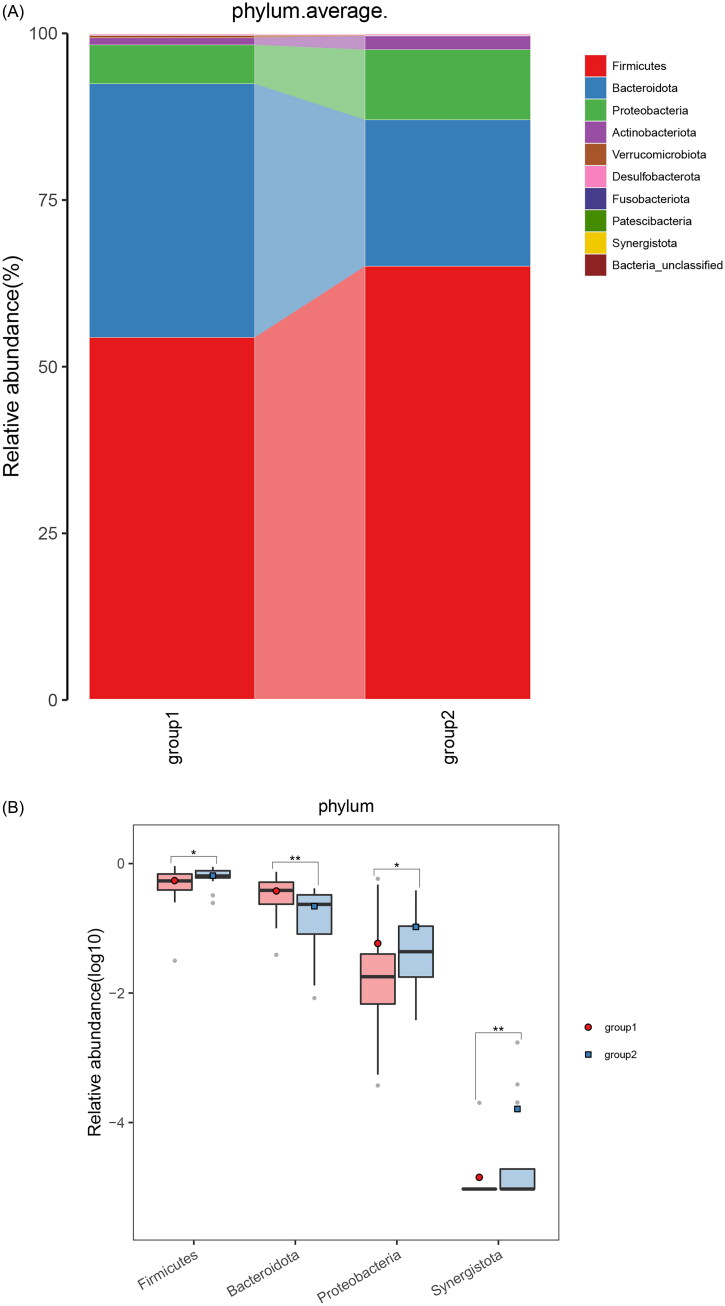

**Figure:**
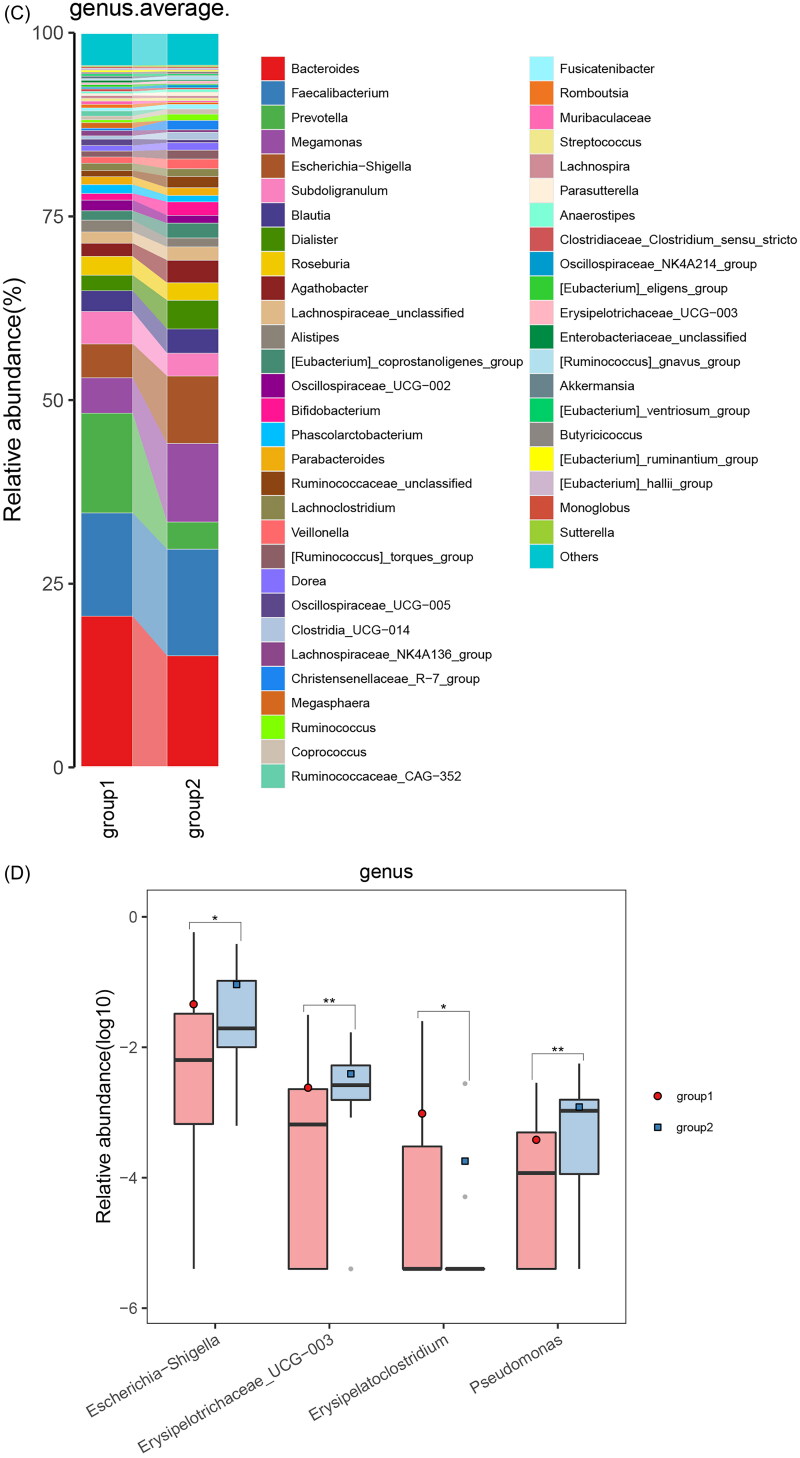

**Figure 4.**
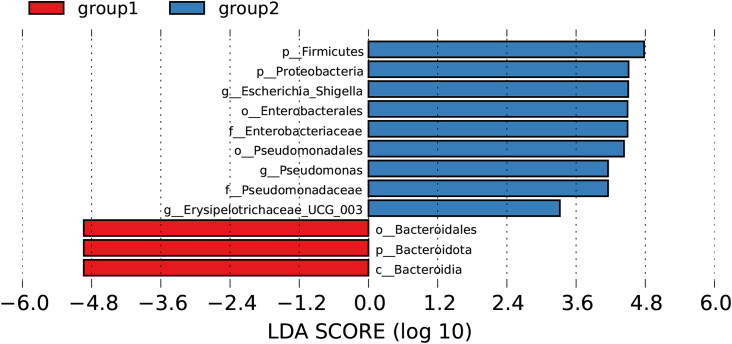
The bar chart highlights key bacterial taxa associated with nonresponders, identified through LDA with a threshold of LDA > 2.5.

The phylogenetic distribution of gut microbiota was differentiated between the two groups using LEfSe analysis. Key bacterial community differences with LDA scores greater than 2.5 were selected. *Escherichia-Shigella* and *Pseudomonas* made the greatest contributions to the differences between responders and nonresponders (all *p* < 0.05; Figure 4).

### 3.4. Identification of key microbial ASVs and diagnostic potential of the bacterial classifier for predicting outcomes in IgA nephropathy

At the ASV level, a random forest model was constructed based on gut microbiota associated with nonresponders to assess the capacity of the fecal microbiome to discriminate between responders and nonresponders. Notably, through five-fold cross-validation, 24 ASVs were identified as key bacterial communities for nonresponders, with *Escherichia-Shigella* and *Pseudomonas* (including ASV1, ASV347, ASV476, and ASV468) showing the greatest significance in the nonresponders classifier based on mean decrease accuracy (Figure 5(A,B)) and mean decrease Gini analyses (Supplemental Figure 2). The progression of disease (POD) classifier for nonresponders achieved an AUC of 0.9103 under the ROC curve (95% CI: 0.83–0.99; *p* < 0.0001; Figure 5(C,D)). These findings suggest that the bacterial classifier may serve as a highly diagnostic, noninvasive tool for predicting treatment response in patients with IgAN.

Figure 5.Diagnostic efficiency of gut bacterial markers to distinguish nonresponders in IgAN patients.

**Figure:**
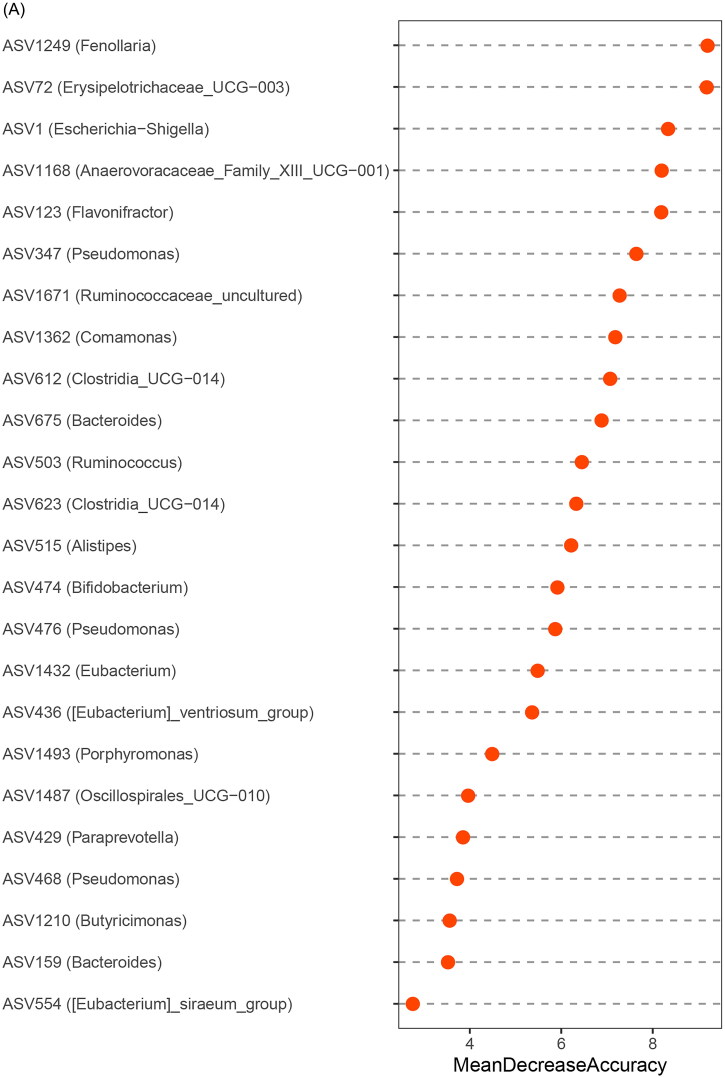

**Figure:**
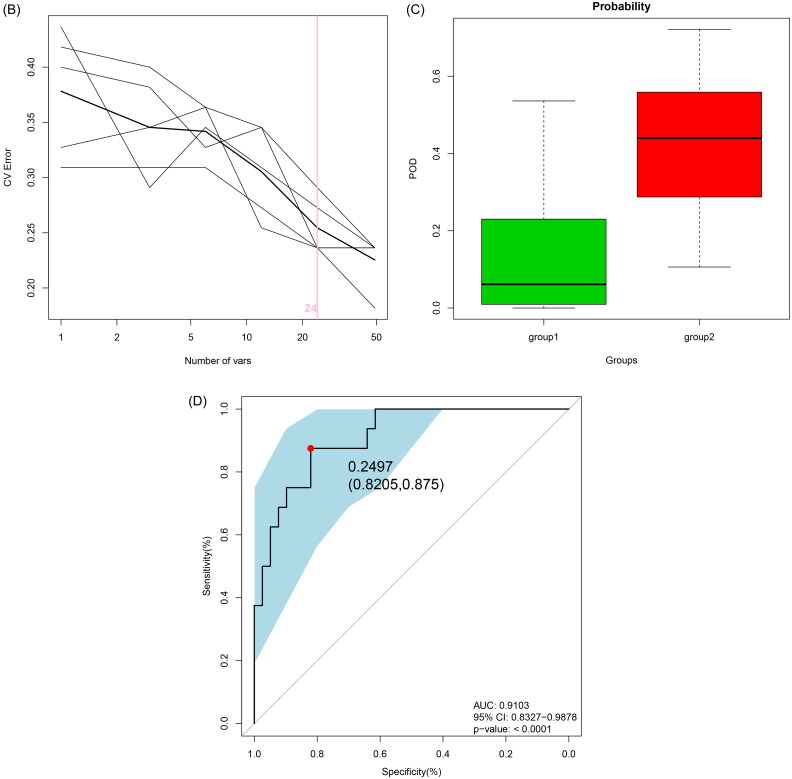

### 3.5. Functional alterations in fecal microbiota of nonresponders in IgA nephropathy

**Figure 6.**
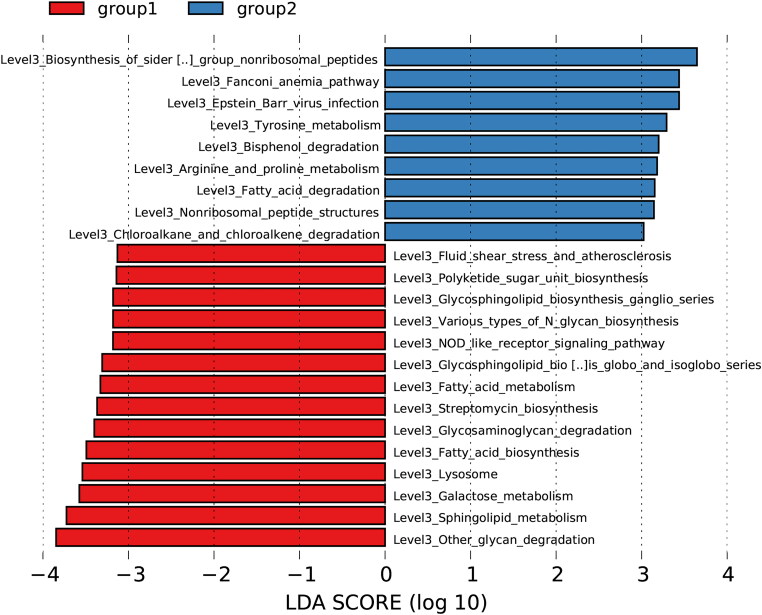
Predicted microbial functional pathways enriched in nonresponders (PICRUSt2 analysis).

We assessed the functional composition profiles by analyzing 16S rRNA sequencing data using PICRUSt2 to explore functional alterations in the fecal microbiota of nonresponders. Based on LDA selection (LDA > 3) and level 3 KEGG pathways, nine predicted microbial functional pathways were significantly enriched in nonresponders (all *p* < 0.05; Figure 6). These pathways were associated with the degradation of hazardous chemical substances, amino acid metabolism (including proline, arginine, and tyrosine metabolism), and fatty acid degradation.

## 4. Discussion

The relationship between gut microbiota disorders and many immune-related diseases has been established in previous studies [18]. In recent years, the role of the gut microbiome in the development and progression of IgAN has increasingly garnered attention and been progressively explored [19]. These studies have demonstrated a strong connection between the gut microbiome and IgAN. However, specific alterations in the gut microbiome of IgAN patients, particularly the distinct differences between those who respond to treatment (responders) and those who do not (nonresponders), have been rarely reported.

Our study provides compelling evidence of gut microbiota dysbiosis in nonresponders to treatment in IgAN patients, underscoring the potential role of microbial imbalances in the disease’s progression and suggesting new therapeutic strategies targeting the microbiome. Specifically, in nonresponders, we observed significant enrichment of *Proteobacteria* and *Firmicutes* which has been proposed as a common signature of microbial dysbiosis and is closely related to the maintenance of host health [20,21]. These taxa, along with opportunistic pathogens such as *Escherichia-Shigella*, *Pseudomonas*, and *Erysipelotrichaceae*, have previously been linked to inflammatory diseases and immune dysregulation. A clinical study conducted at Xijing Hospital [22] reported that, compared to the healthy control group, IgAN patients exhibited an increased proportion of *Proteobacteria* (phylum) in the gut, along with elevated levels of *Escherichia-Shigella* (genus). Additionally, correlation analysis revealed a significant negative association between the abundance of *Clostridium* (genus) and serum albumin levels. *E. coli*, as a member of *Escherichia-Shigella*, acquired through intraperitoneal administration, contributed to the deposition of IgA and C3 in mice mesangial cells. *Escherichia-Shigella* could initiate various pathophysiological cascades by producing lipopolysaccharide (LPS). A high level of LPS activates the NF-κB pathway, triggering the production of IL-6 and interferon-α [23]. A significant expansion of the *Escherichia-Shigella* genus was observed by Zhao et al. in untreated IgAN patients. This expansion was reversed in patients in clinical remission following immunosuppressive therapy [24]. Meanwhile, study suggested that recurrent or chronic exposure to *Pseudomonas* may increase complications in patients with type 1 diabetes (T1D) by enhancing susceptibility to chronic inflammation [25]. However, bacteria in the *Pseudomonas* genus are opportunistic pathogens. Studies have shown an increased abundance of *Pseudomonas* in patients with irritable bowel syndrome (IBS), suggesting its potential role in the onset of IBS [26,27]. Other research has demonstrated that a diet rich in marine prebiotic fucoidans can promote the growth of beneficial *Bacteroides* populations by inhibiting the interaction between secreted virulence factors (TpsA/CdiA) and mucins, leading to earlier recovery from intestinal dysbiosis and the decolonization of *Pseudomonas* from the gut [28]. Moreover, Lauriero et al. found through correlation analysis that serum IgA1 levels and IgA1 glomerular deposition were positively correlated with the *Erysipelothrix* genera, suggesting that the *Erysipelotrichaceae* genus may be involved in the pathogenesis and progression of IgAN [29].

This study highlights the potential role of the gut microbiota in the pathogenesis of IgAN, emphasizing the relationship between microbial functional pathways and metabolic dysregulation in nonresponders. In our analysis of microbial functional pathways, we identified significant enrichment in pathways related to the degradation of hazardous chemicals, amino acid metabolism (including proline, arginine, and tyrosine metabolism), and fatty acid degradation in nonresponders. These findings are particularly intriguing as they suggest that alterations in microbial function may contribute to the metabolic dysregulation observed in IgAN patients. The gut microbiota may influence renal function through the production of metabolites that affect the kidney’s inflammatory response or fibrosis [24]. For instance, changes in the levels of proline and arginine could impact collagen synthesis and fibrosis in the kidney [30], which is a hallmark of IgAN progression. Additionally, alterations in fatty acid degradation may impact inflammatory pathways, as certain fatty acid metabolites are known to modulate immune responses and contribute to systemic inflammation [31]. Enhanced amino acid metabolism led to a reduction in amino acid content, disrupting protein synthesis and affecting various bodily functions, such as gene transcription, the cell division cycle, and the tricarboxylic acid cycle [32]. Studies have also linked spleen tyrosine kinase to mesangial hyperplasia in IgAN, with one potential underlying mechanism involving the activation of NF-κB and p-42/p-44 MAPK signaling pathways, mediated by spleen tyrosine kinase [33]. These functional alterations suggest that the gut microbiota not only affects immune regulation but also plays a role in metabolic pathways that influence kidney health. Targeting these pathways through microbiome-modulating interventions could provide new therapeutic strategies to mitigate disease progression and improve patient outcomes. Rifaximin, a non-absorbable oral antibiotic, helps restore the host’s gut microbiome by promoting the growth of beneficial bacteria. It can also downregulate Gd-IgA1 by inhibiting the TLR-4/NF-kB signaling pathway, subsequently reducing B cell activating factor of the TNF family (BAFF). In mice, this led to decreased urinary protein, hIgA1-SCD89, mIgG-hIgA1 complex levels, and IgA mesangial deposition, suggesting that rifaximin may be a potential treatment for IgAN [34]. Probiotics and prebiotics have also been explored as treatment strategies for IgAN through the regulation of the intestinal microbiota and host immune responses [35]. Tan et al. reported that fecal microbiota dysbiosis in IgAN could be mitigated by administering probiotics rich in Bifidobacterium. Both probiotics and their SCFA metabolites were shown to attenuate clinical indexes and renal pathology manifestations of IgAN by modulating the NLRP3/ASC/Caspase 1 signaling pathway [36].

Our study highlights the potential of gut microbiota profiling as a powerful tool for predicting treatment outcomes in IgAN, paving the way for more personalized and noninvasive approaches to managing the disease. Our analysis of gut microbiota and the development of a predictive model for IgAN outcomes based on key microbial taxa represent a significant advancement in understanding the role of the microbiome in disease progression. Research to date has demonstrated that the composition of the gut microbiome influences clinical response to therapy [37–39]. Using a random forest model, we identified 24 key ASVs that could distinguish nonresponders from responders with high accuracy, as demonstrated by the AUC value of 0.9103 under the ROC curve. *Escherichia-Shigella* and *Pseudomonas* were identified as the most significant contributors to this bacterial classifier, underlining their role in the disease process. The diagnostic potential of this bacterial classifier is substantial. By using gut microbiota profiles as a noninvasive biomarker, clinicians could more accurately predict which IgAN patients are at high risk of disease progression and who would benefit most from immunosuppressive therapies. This could lead to more personalized treatment regimens and reduce the reliance on invasive procedures such as kidney biopsies. Additionally, The ability to predict treatment response based on gut microbiota offers a promising step toward personalized medicine in IgAN, where tailored therapies could optimize outcomes and reduce unnecessary side effects associated with systemic immunosuppressive therapies.

A key strength of our study is the inclusion of early-stage IgAN patients (eGFR > 60 mL/min/1.73 m^2^), minimizing potential confounding from advanced CKD. Since gut microbiota undergoes significant alterations in end-stage renal disease due to uremic toxin accumulation [40], excluding moderate to severe CKD patients ensures a clearer focus on IgAN-related microbiota changes. This criterion also improves cohort homogeneity, reducing variability and enhancing reliability. Future studies should explore microbiota alterations across different CKD stages to further validate our findings.

While this study provides valuable insights into the role of gut microbiota in IgAN, certain limitations should be noted. First, the small sample size may affect the generalizability of the findings, necessitating larger, multi-center studies to validate microbial biomarkers across diverse populations. Second, although all fecal samples were collected before immunosuppressive therapy, minimizing its immediate impact, microbial changes over time cannot be ruled out. Longitudinal sampling should be considered in future research. Finally, the absence of a healthy control group limits broader comparisons. While our focus was on identifying microbiota-based predictors of treatment response, future studies should incorporate healthy controls for a more comprehensive analysis.

In conclusion, our study highlights the gut microbiota’s crucial role in IgAN progression and treatment response. The identified microbial biomarkers, particularly *Pseudomonas* and *Escherichia-Shigella*, offer promising potential for noninvasive diagnosis and personalized therapy. Future research should focus on validating these findings and exploring microbiome-based treatments to improve clinical outcomes for IgAN patients.

## Supplementary Material

Figures.zip

## Data Availability

The datasets used and analyzed during the current study available from the corresponding author on reasonable request.
