# Supplementary figures and images for: Gut microbiota as a predictive tool for outcomes in IgA nephropathy
Source: Ren Fail. 2025 Jul 9;47(1):2514184. doi: 10.1080/0886022X.2025.2514184 (PMC12243011; doi:10.1080/0886022X.2025.2514184)

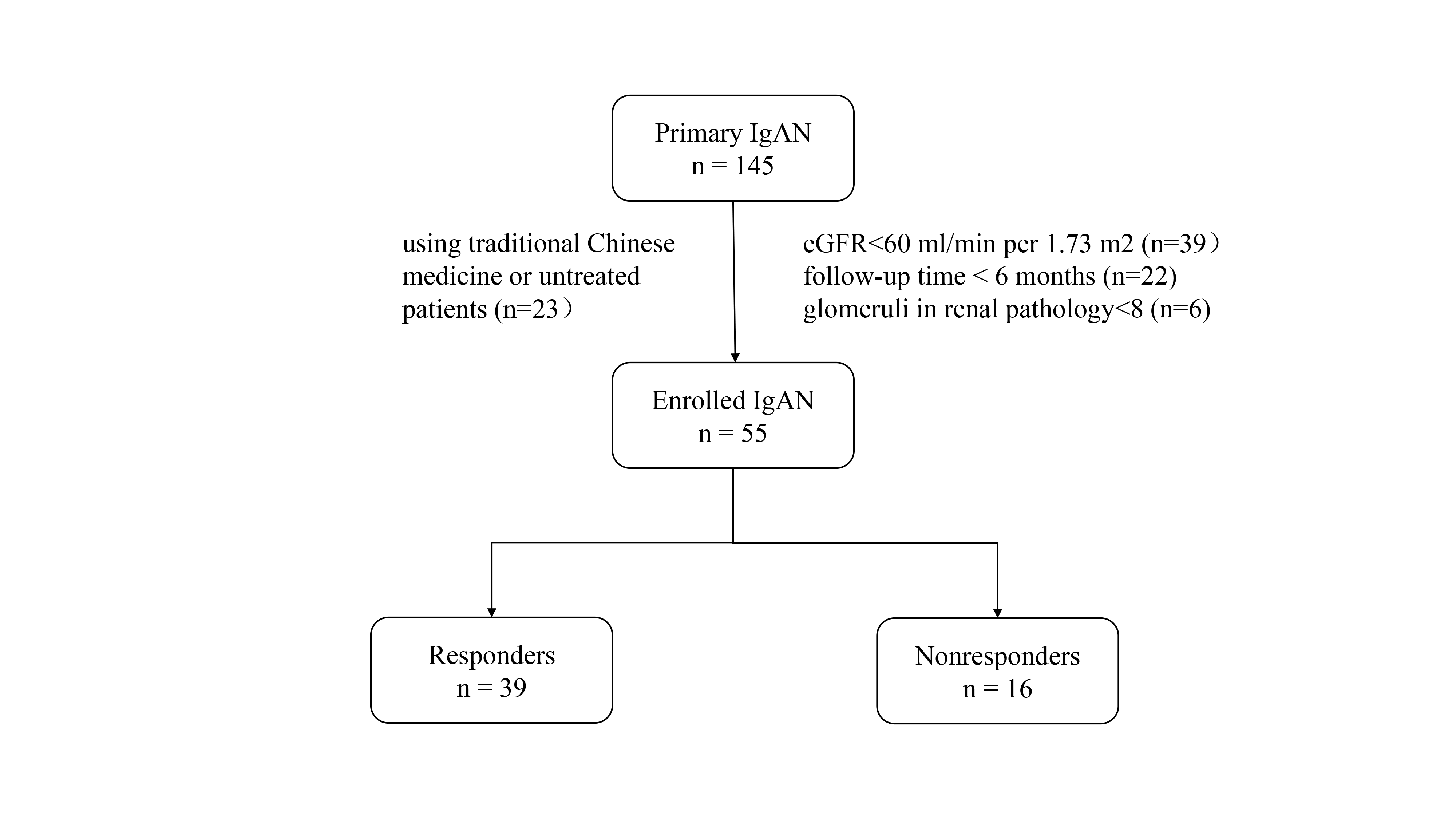

Supplement: Figures.zip [file IRNF_A_2514184_SM1332.zip › Figures/Figure 1.tif]

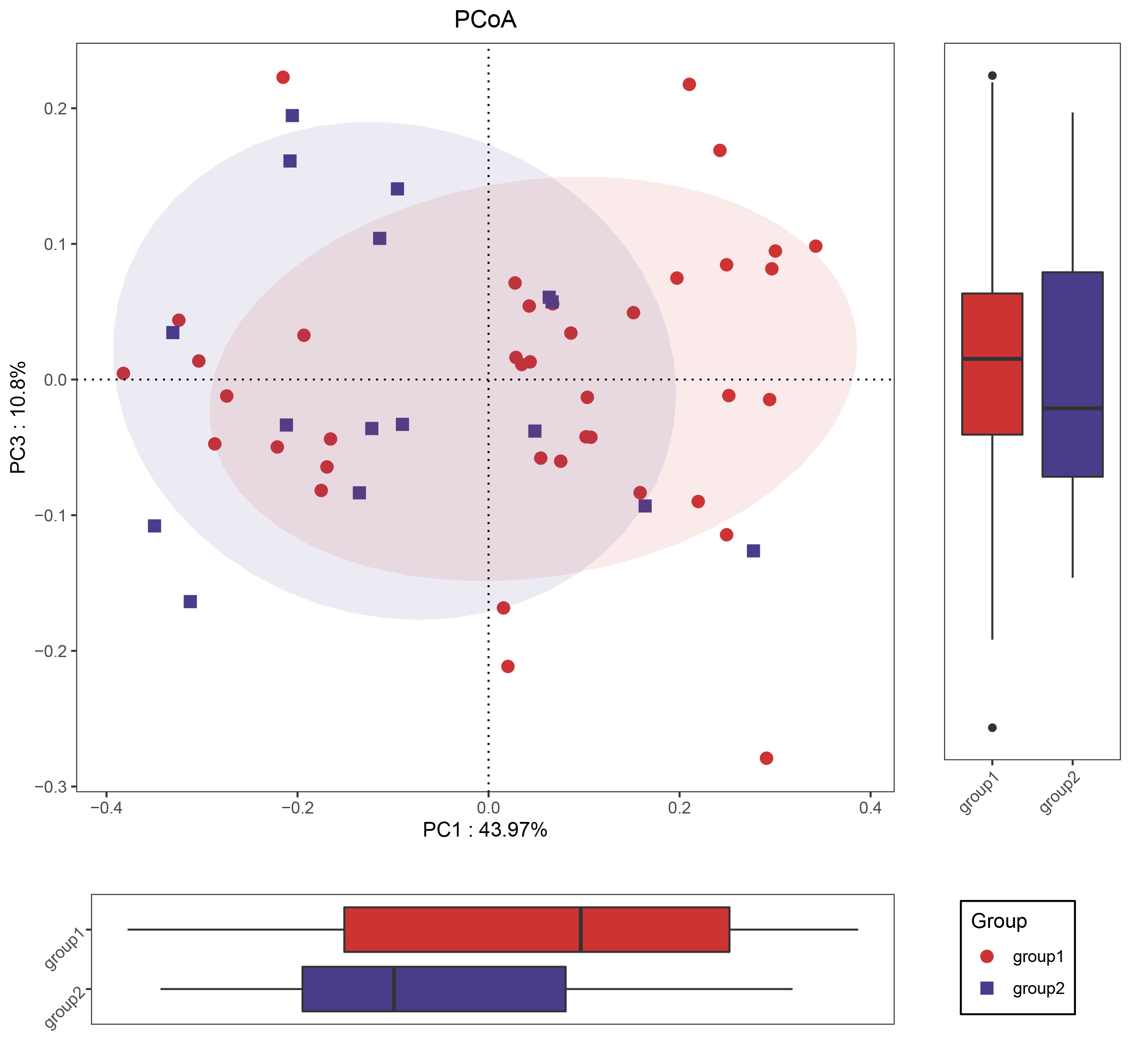

Supplement: Figures.zip [file IRNF_A_2514184_SM1332.zip › Figures/Figure 2A.tif]

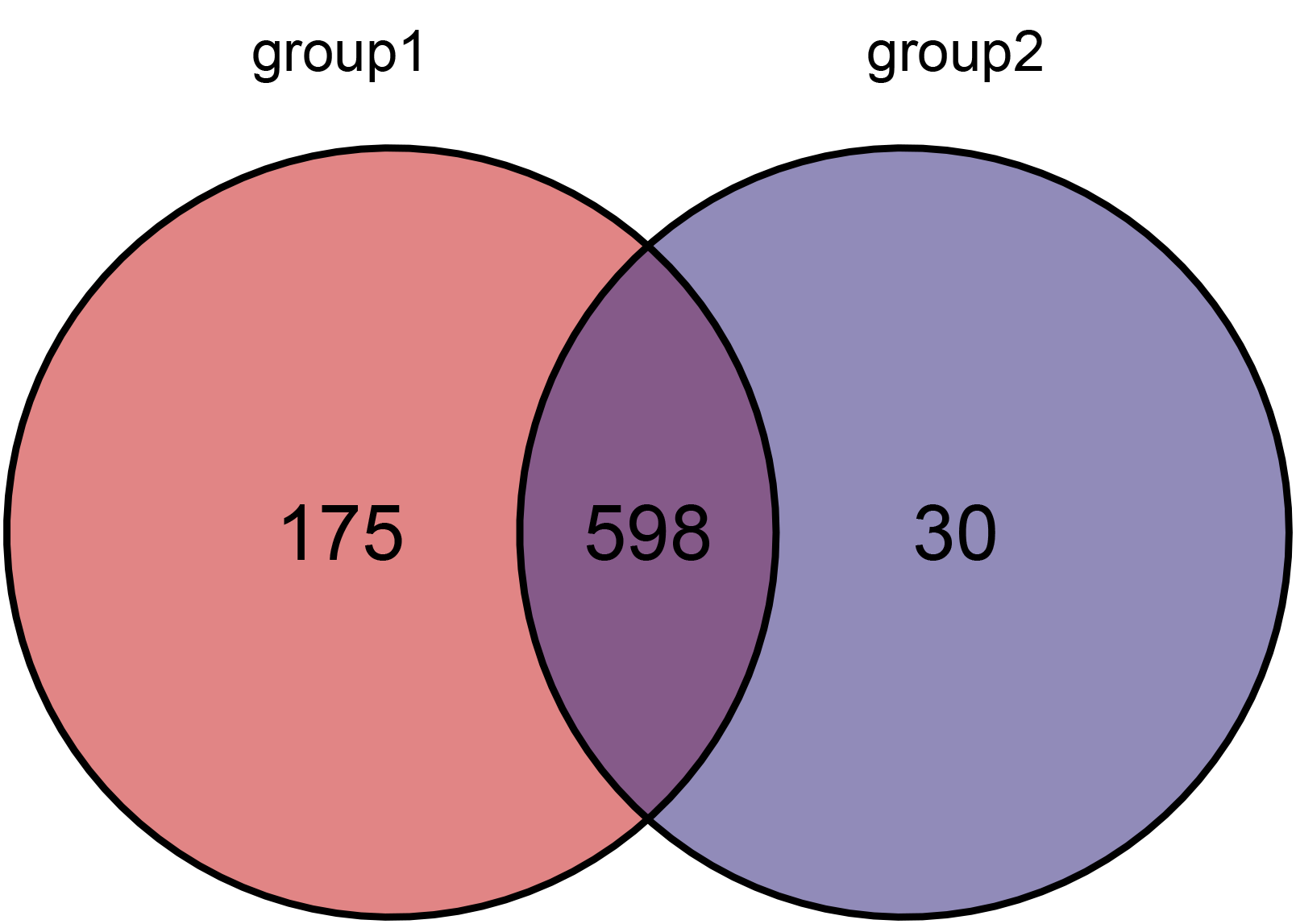

Supplement: Figures.zip [file IRNF_A_2514184_SM1332.zip › Figures/Figure 2B.tif]

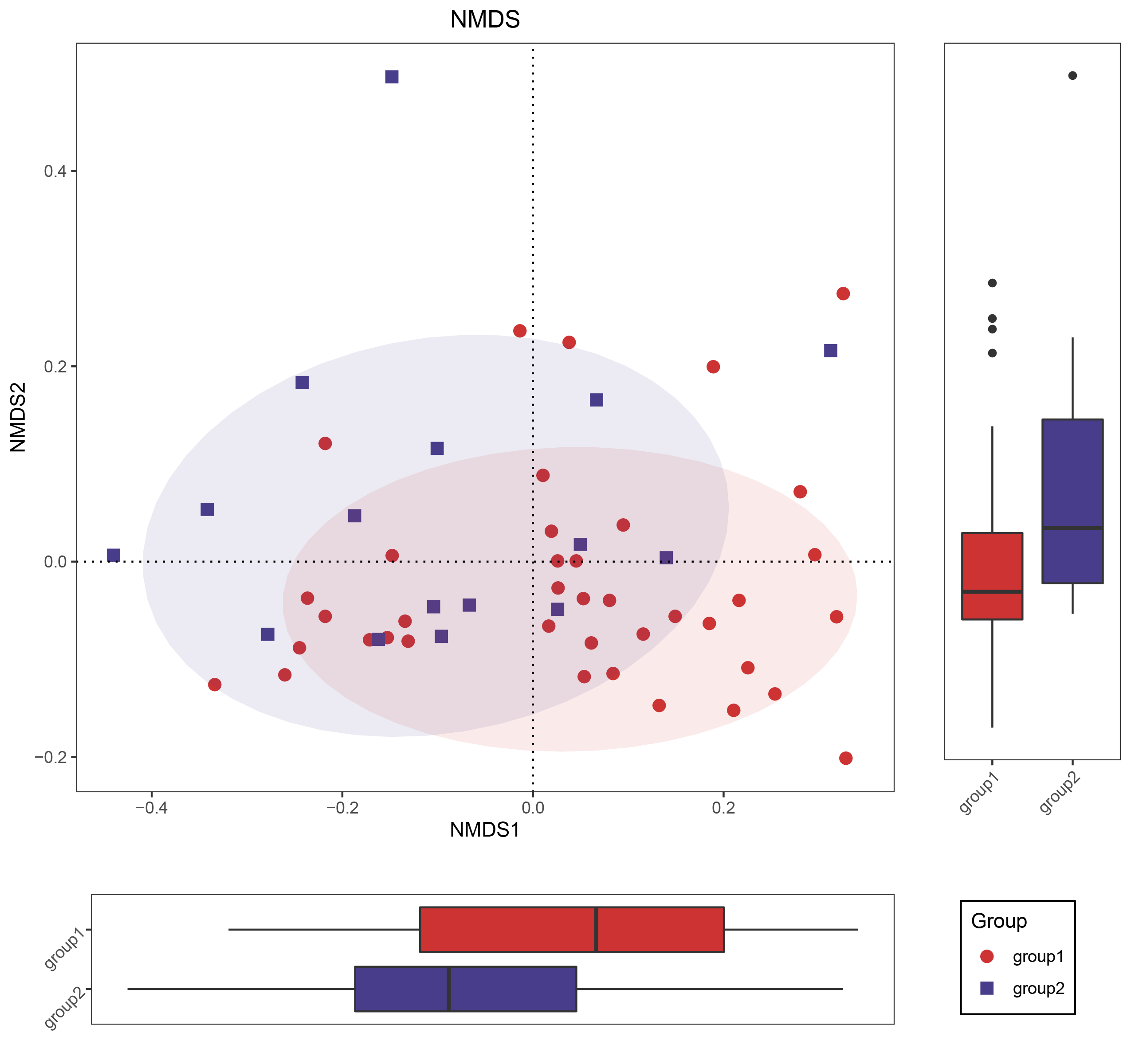

Supplement: Figures.zip [file IRNF_A_2514184_SM1332.zip › Figures/Figure 2C.tif]

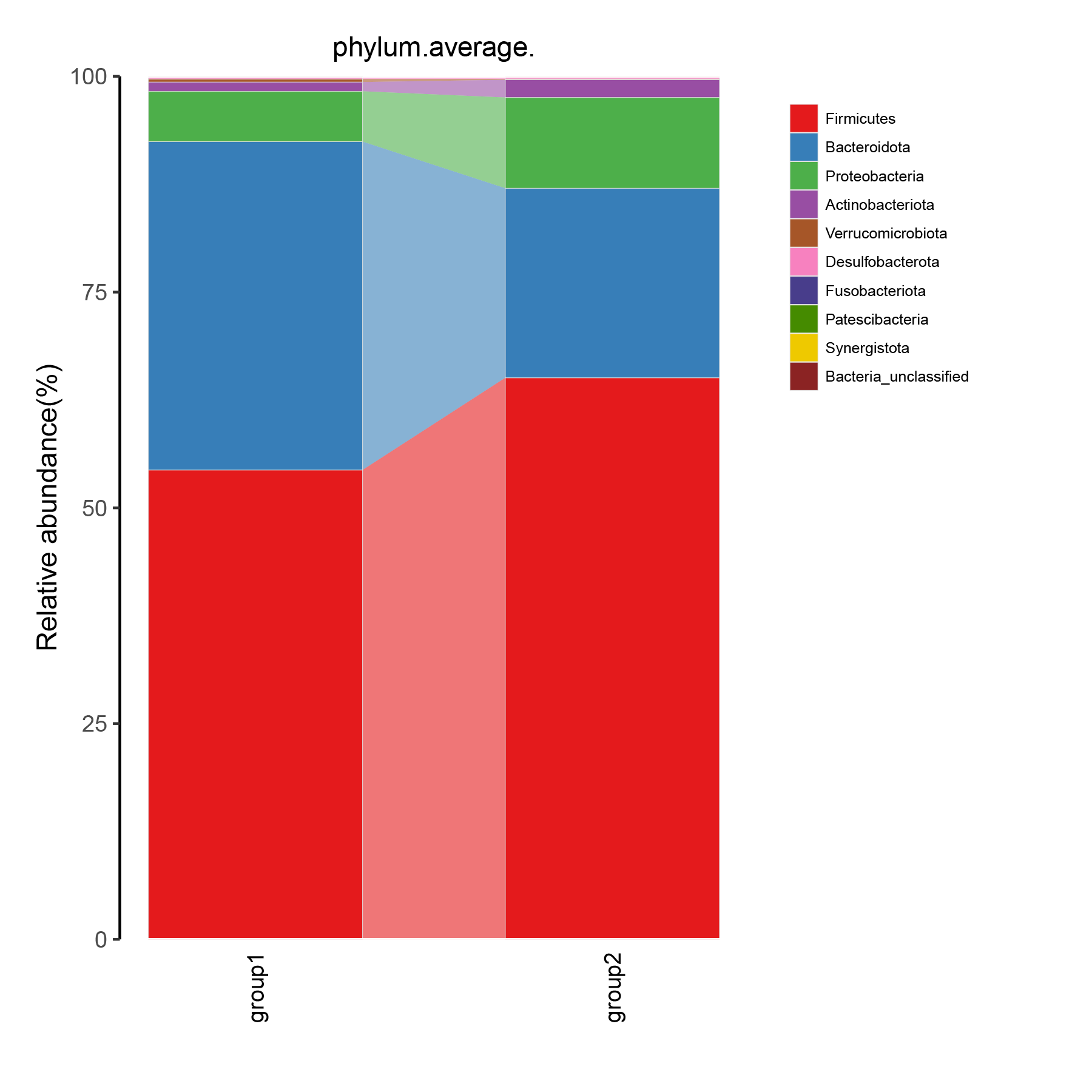

Supplement: Figures.zip [file IRNF_A_2514184_SM1332.zip › Figures/Figure 3A.tif]

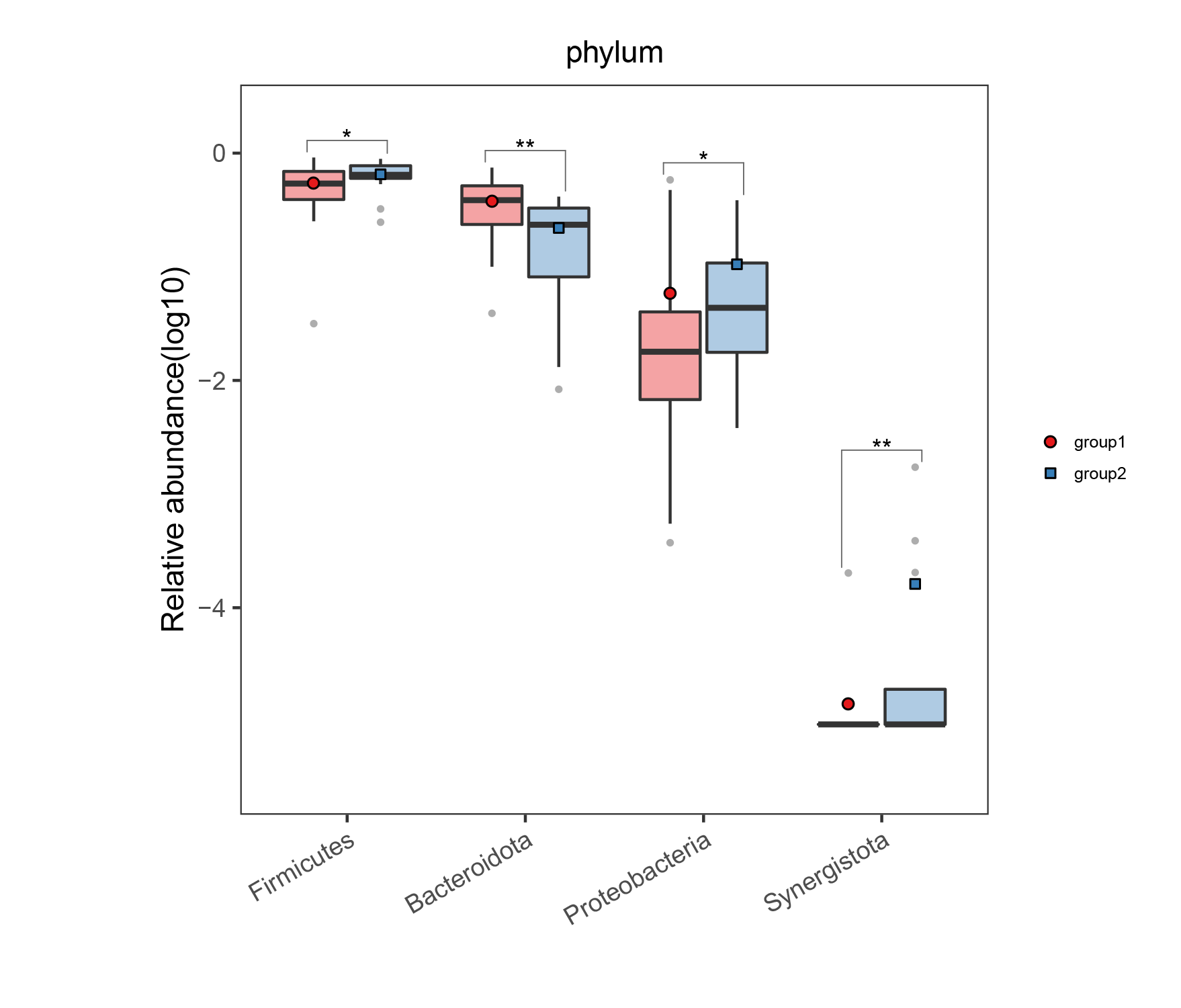

Supplement: Figures.zip [file IRNF_A_2514184_SM1332.zip › Figures/Figure 3B.tif]

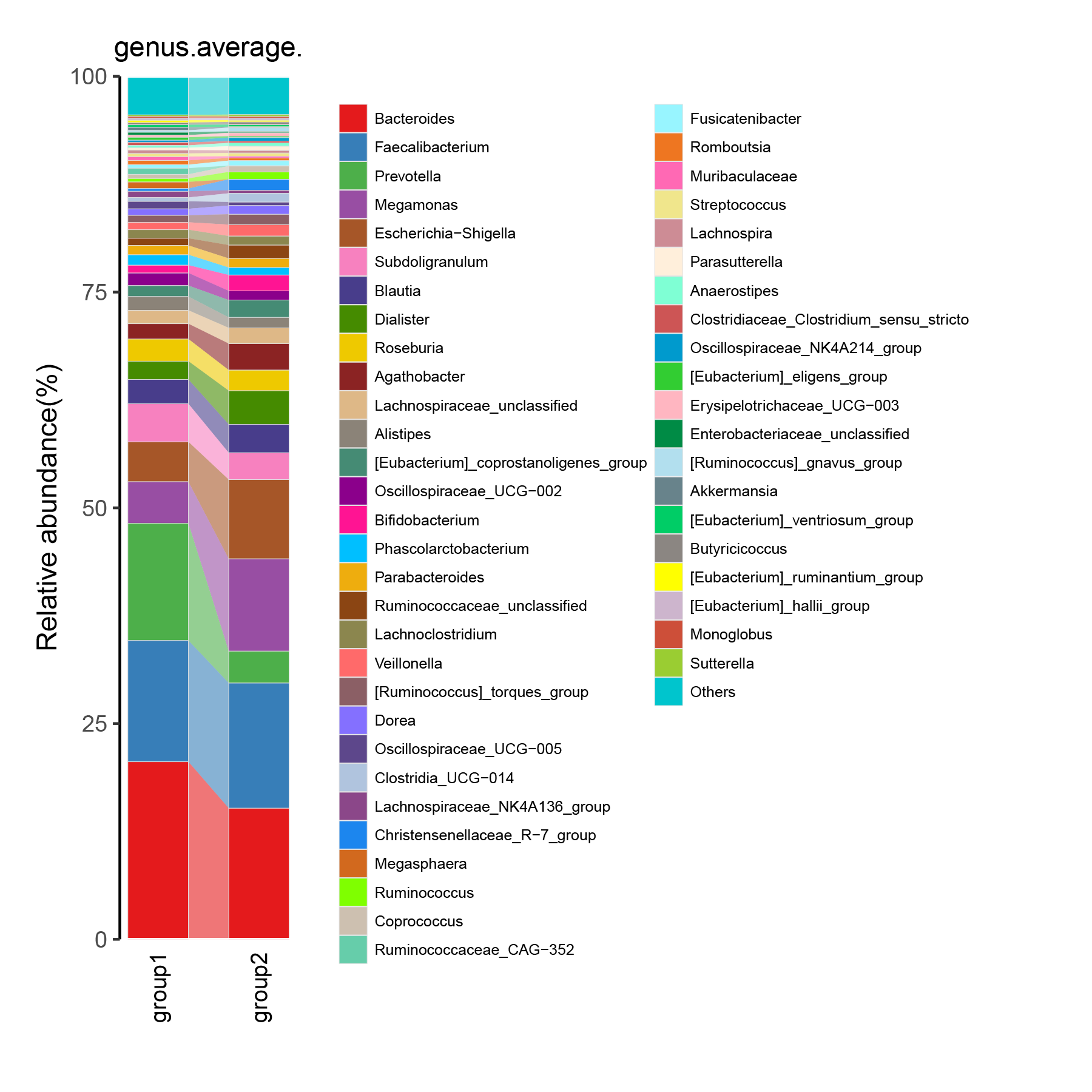

Supplement: Figures.zip [file IRNF_A_2514184_SM1332.zip › Figures/Figure 3C.tif]

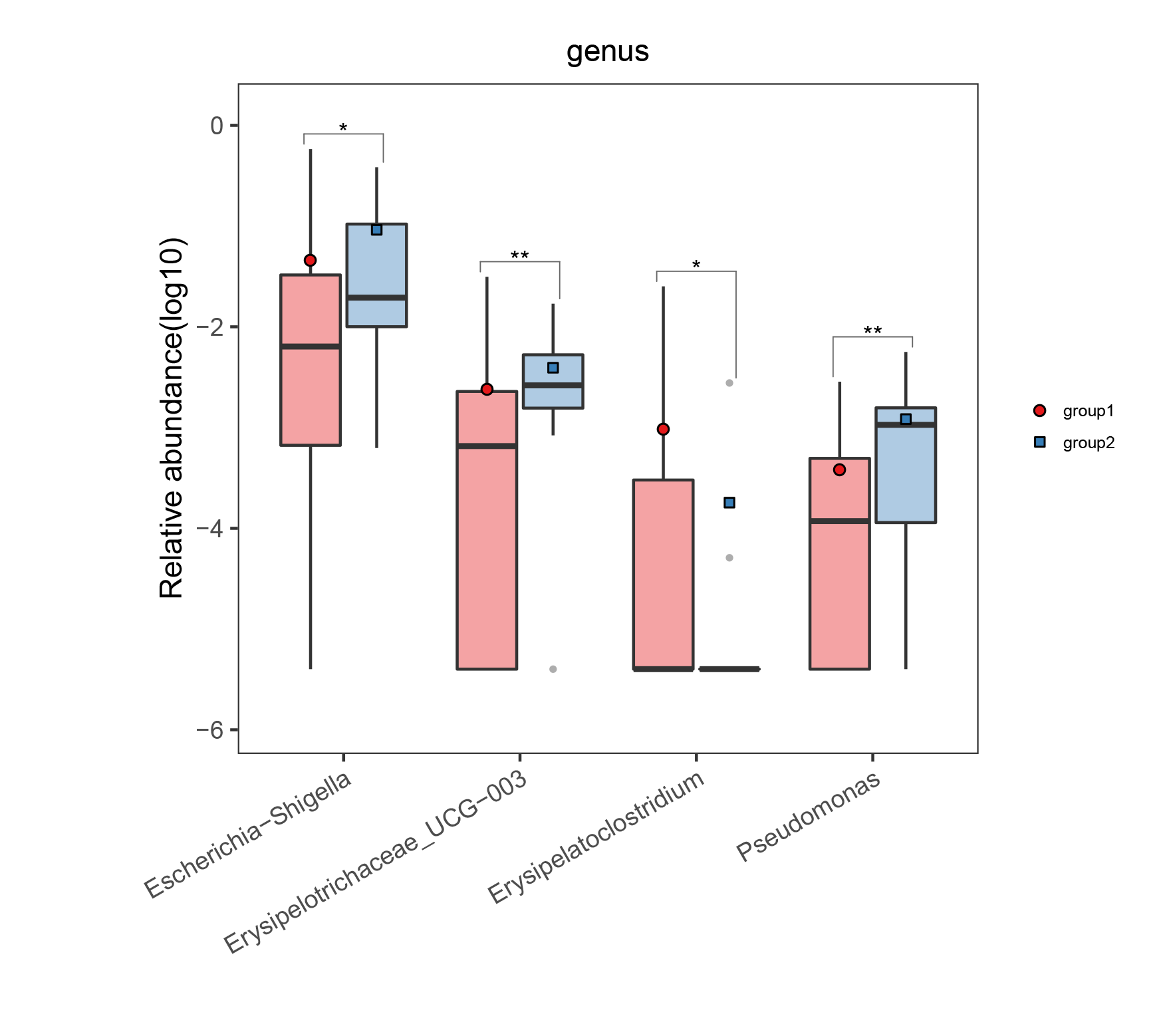

Supplement: Figures.zip [file IRNF_A_2514184_SM1332.zip › Figures/Figure 3D.tif]

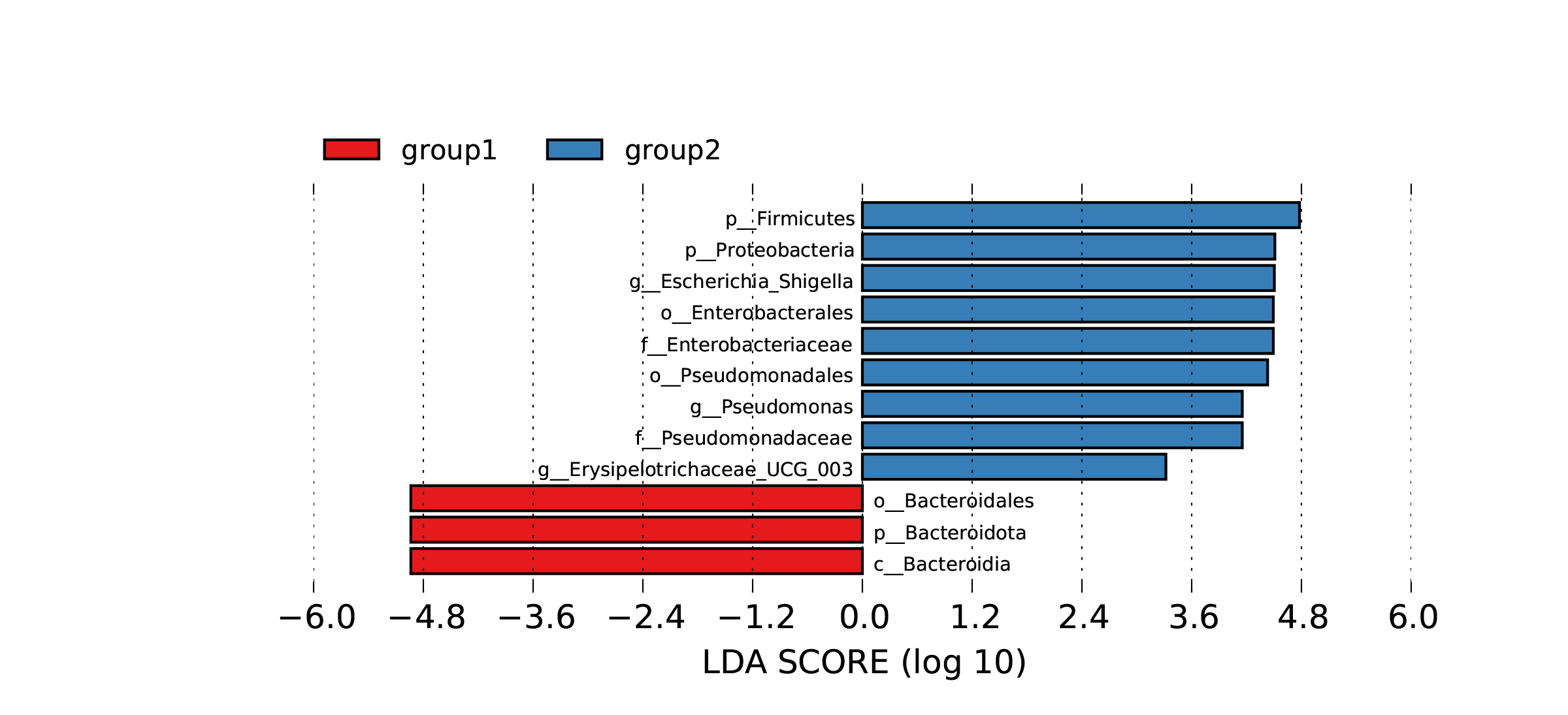

Supplement: Figures.zip [file IRNF_A_2514184_SM1332.zip › Figures/Figure 4.tif]

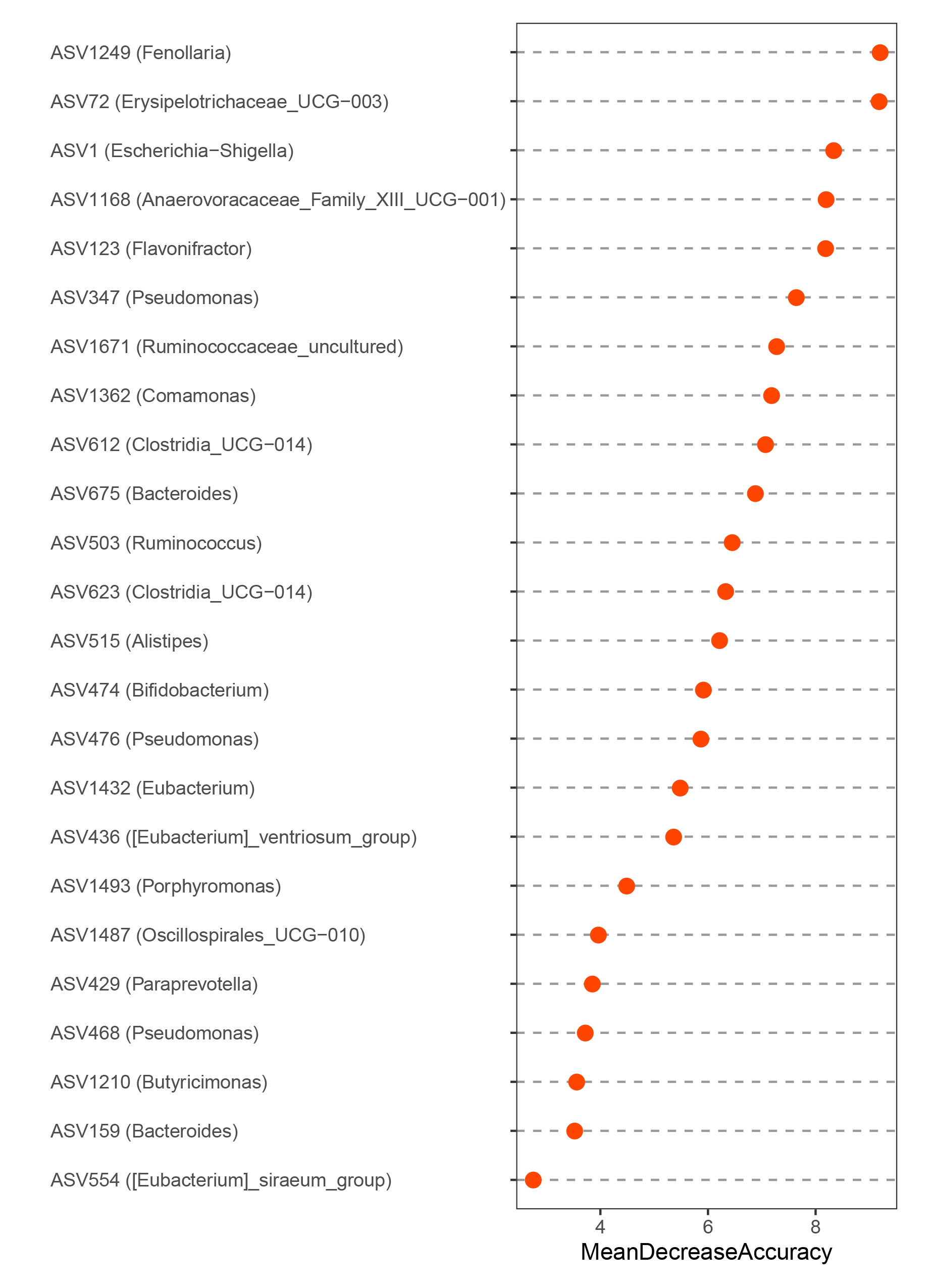

Supplement: Figures.zip [file IRNF_A_2514184_SM1332.zip › Figures/Figure 5A.tif]

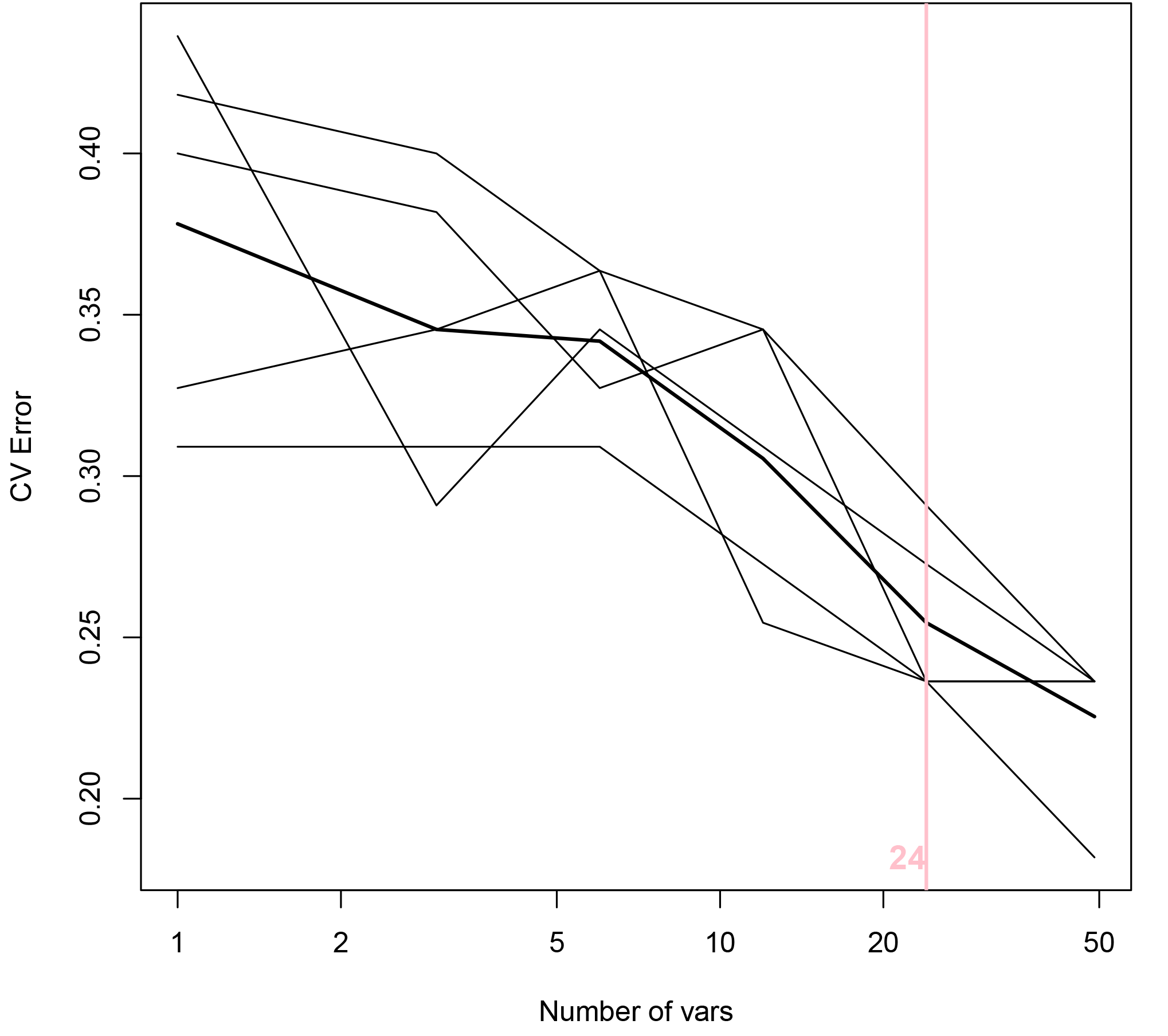

Supplement: Figures.zip [file IRNF_A_2514184_SM1332.zip › Figures/Figure 5B.tif]

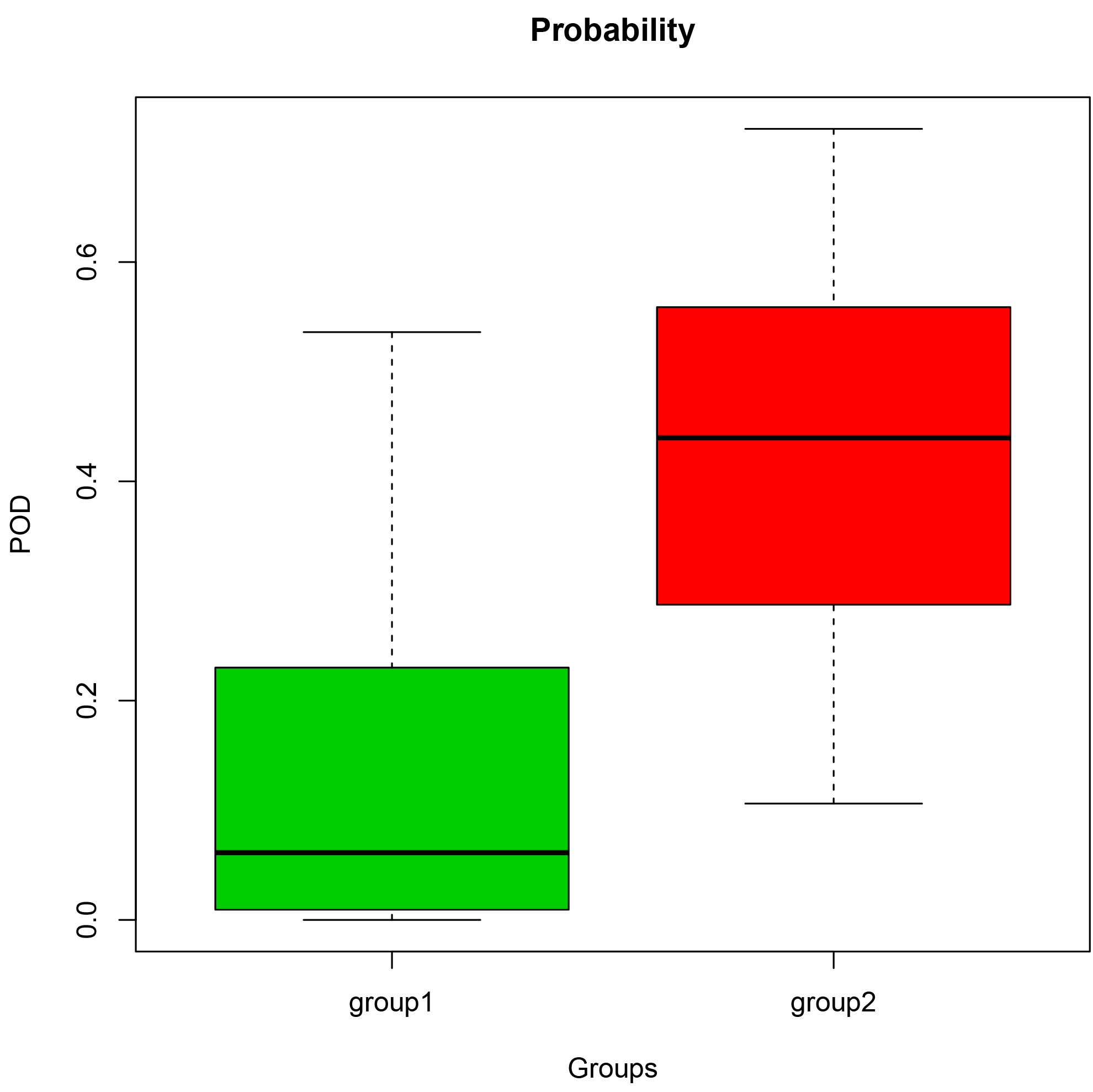

Supplement: Figures.zip [file IRNF_A_2514184_SM1332.zip › Figures/Figure 5C.tif]

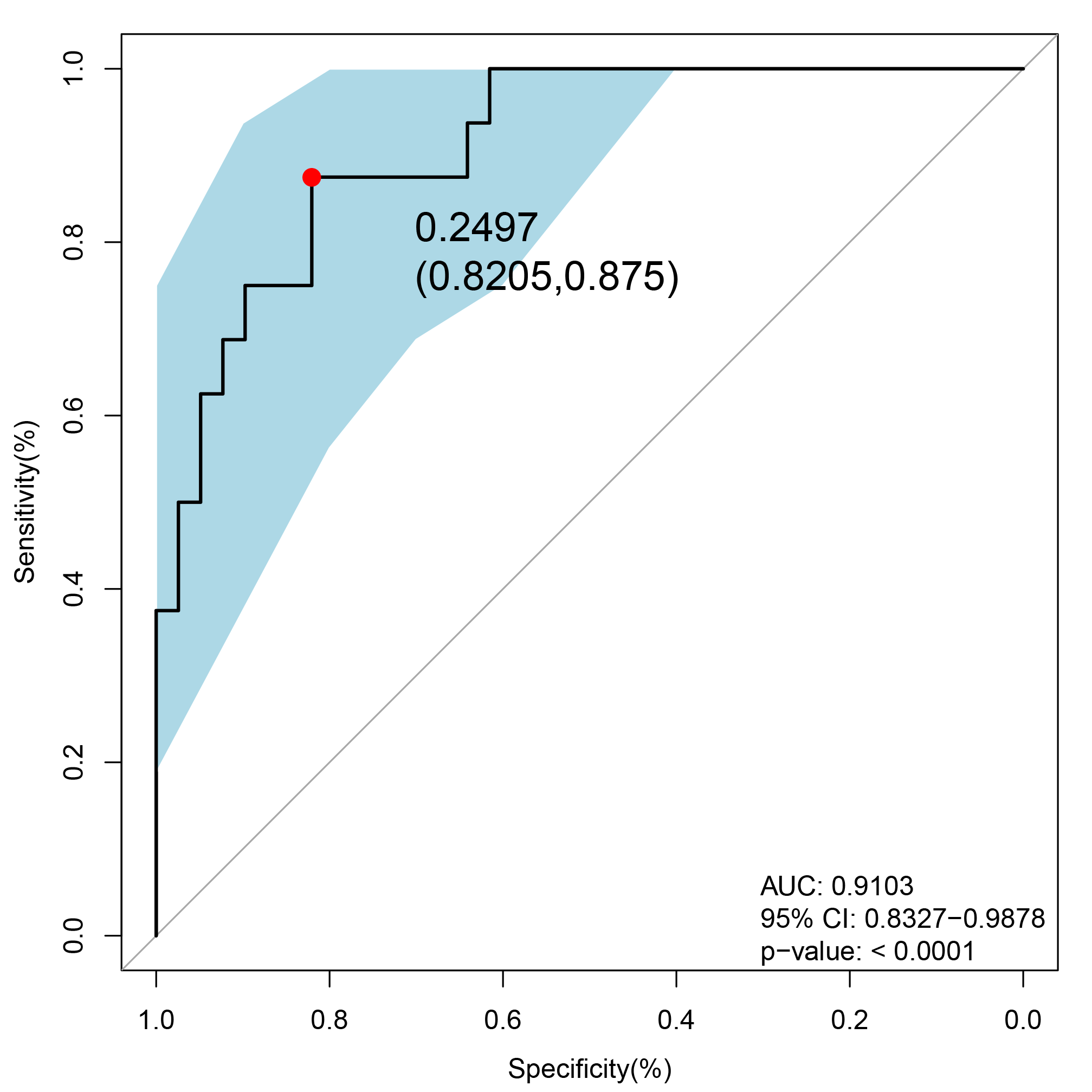

Supplement: Figures.zip [file IRNF_A_2514184_SM1332.zip › Figures/Figure 5D.tif]

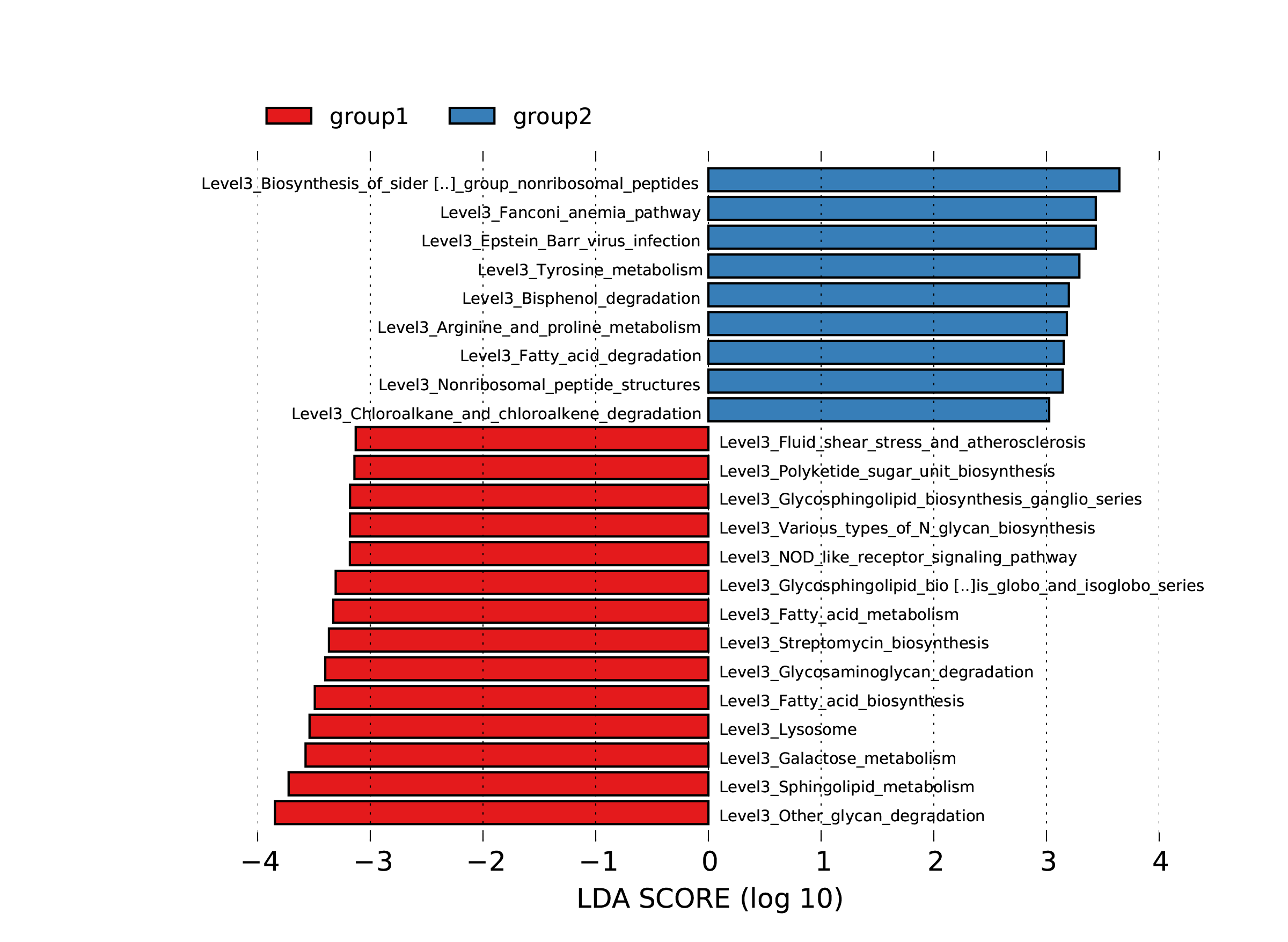

Supplement: Figures.zip [file IRNF_A_2514184_SM1332.zip › Figures/Figure 6.tif]

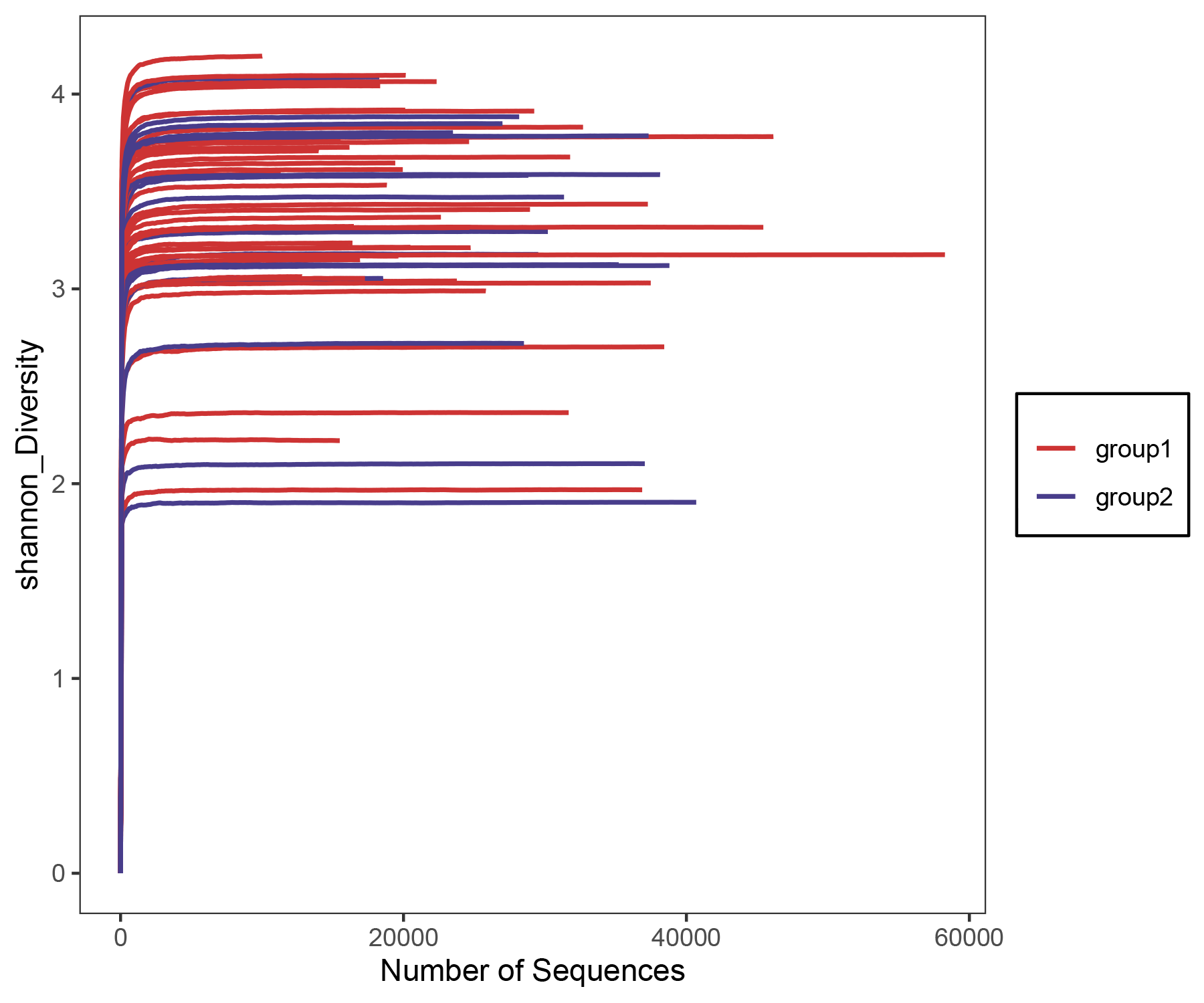

Supplement: Figures.zip [file IRNF_A_2514184_SM1332.zip › Figures/Supplemental Figure 1A.tif]

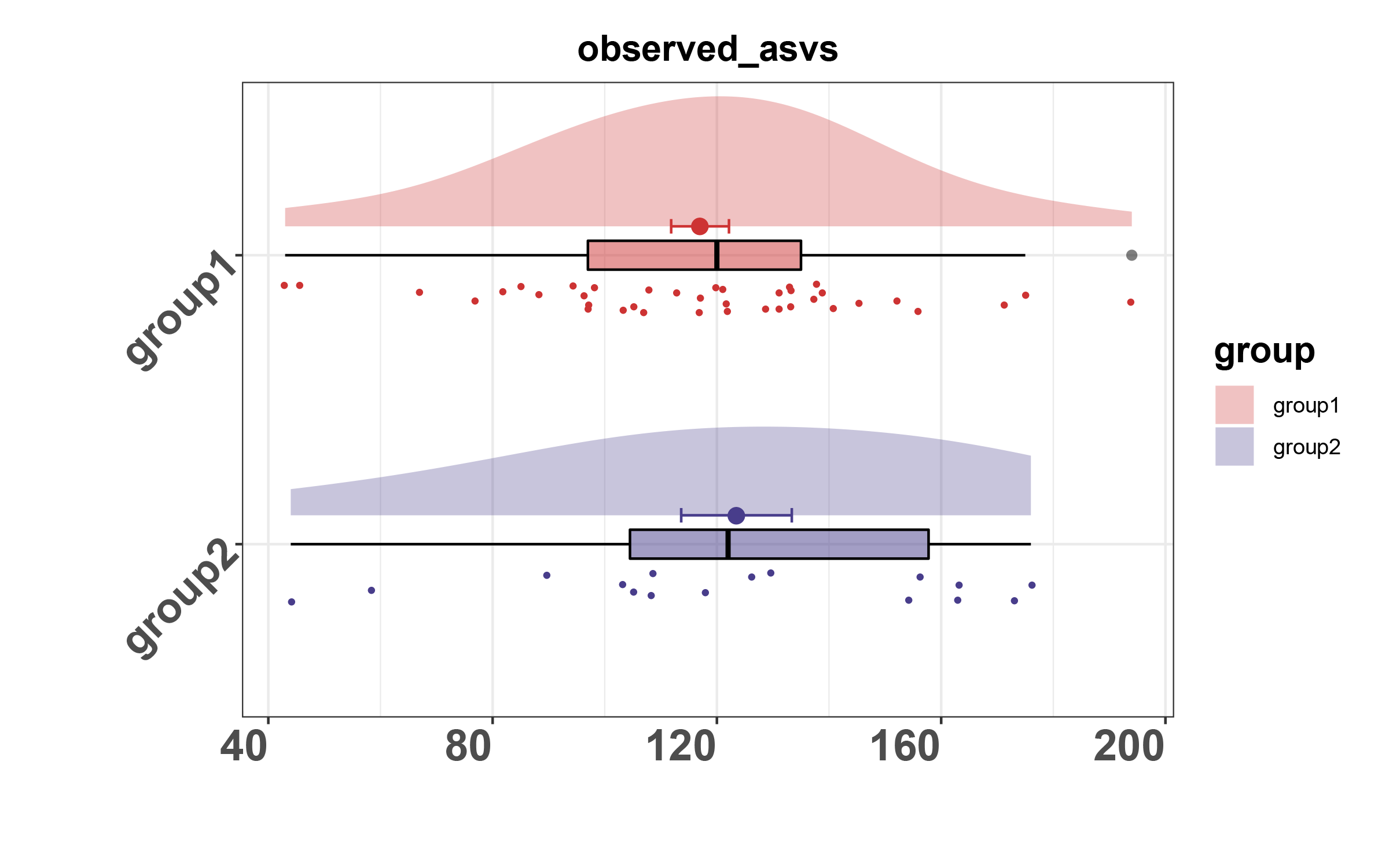

Supplement: Figures.zip [file IRNF_A_2514184_SM1332.zip › Figures/Supplemental Figure 1B.tif]

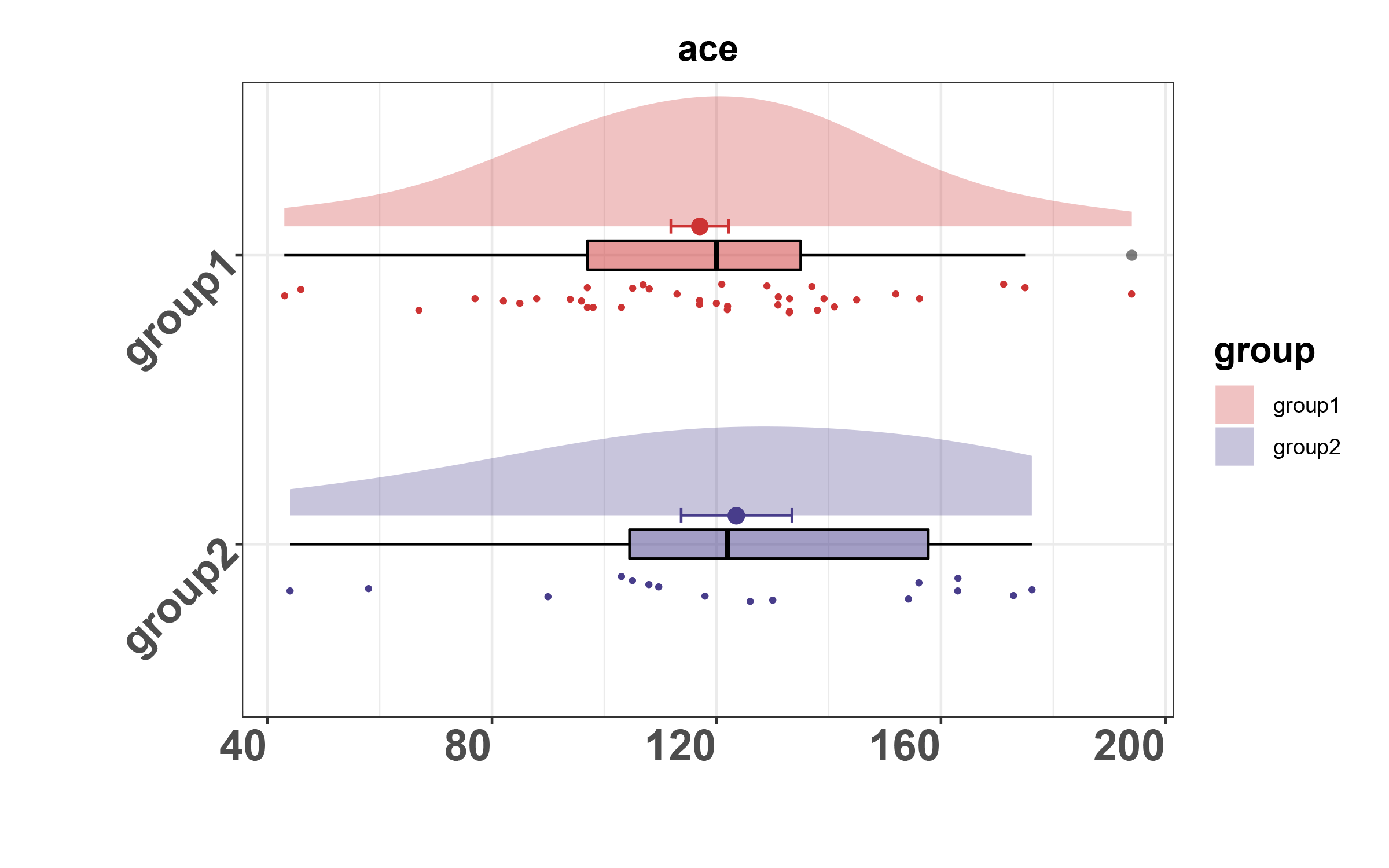

Supplement: Figures.zip [file IRNF_A_2514184_SM1332.zip › Figures/Supplemental Figure 1C.tif]

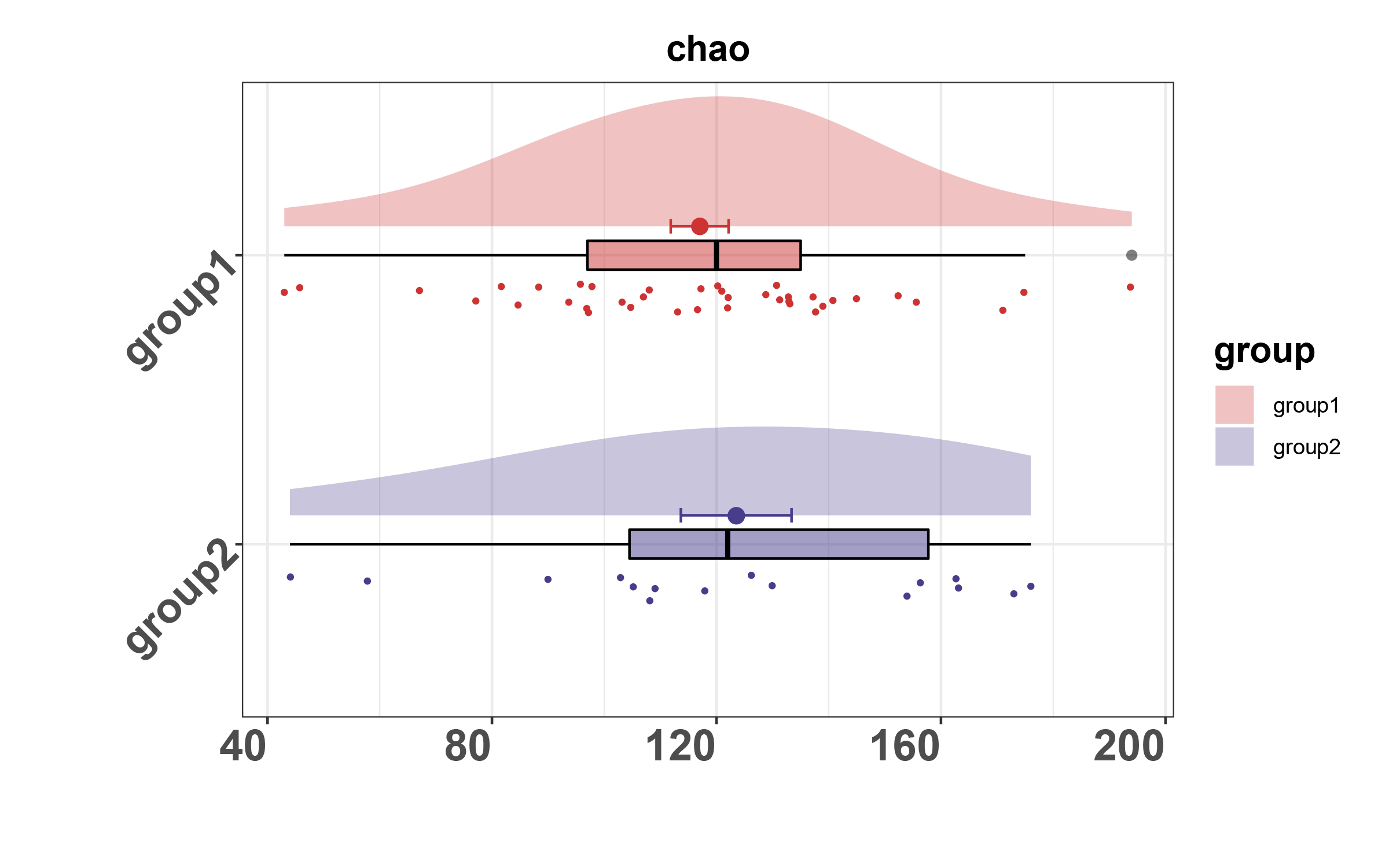

Supplement: Figures.zip [file IRNF_A_2514184_SM1332.zip › Figures/Supplemental Figure 1D.tif]

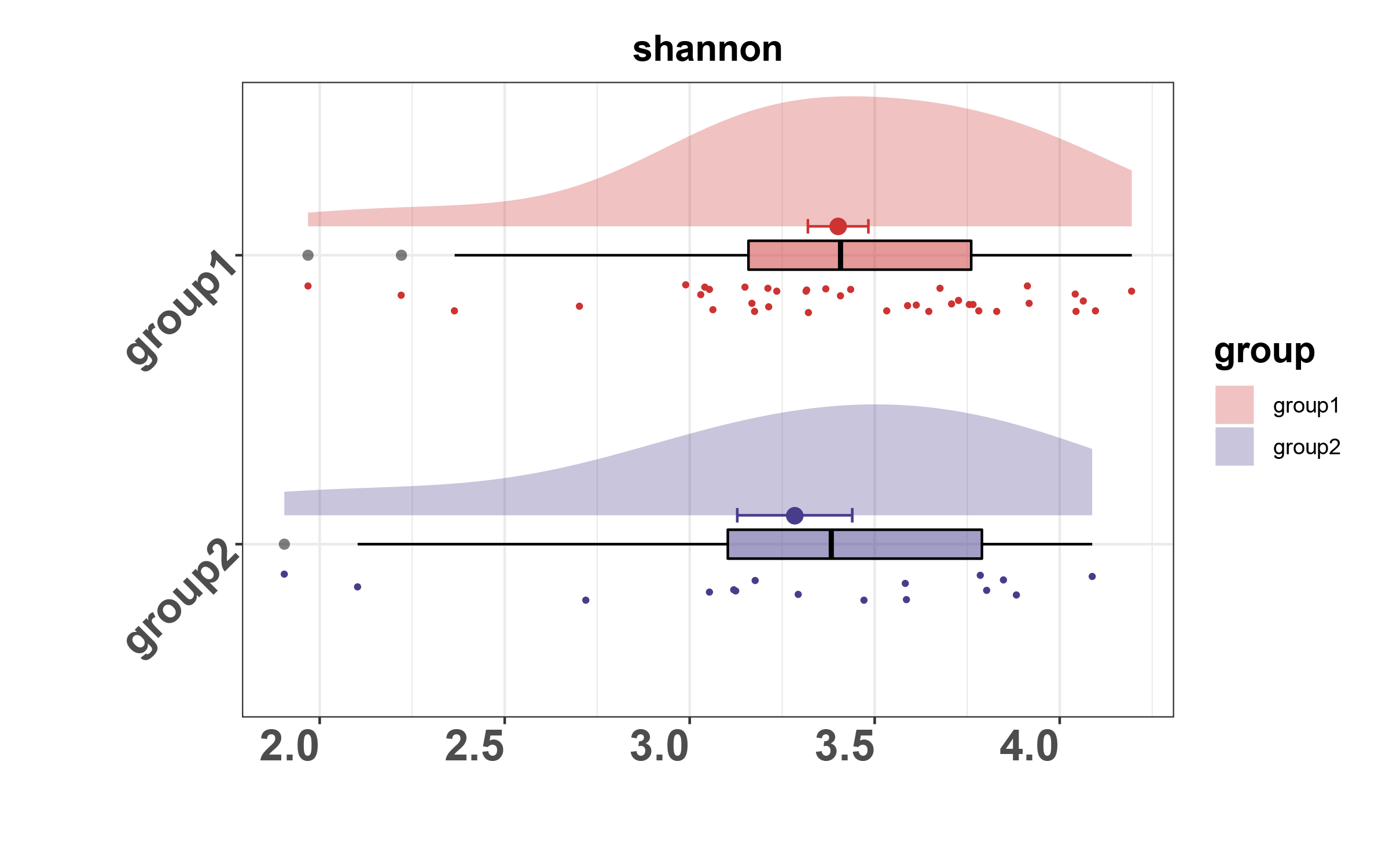

Supplement: Figures.zip [file IRNF_A_2514184_SM1332.zip › Figures/Supplemental Figure 1E.tif]

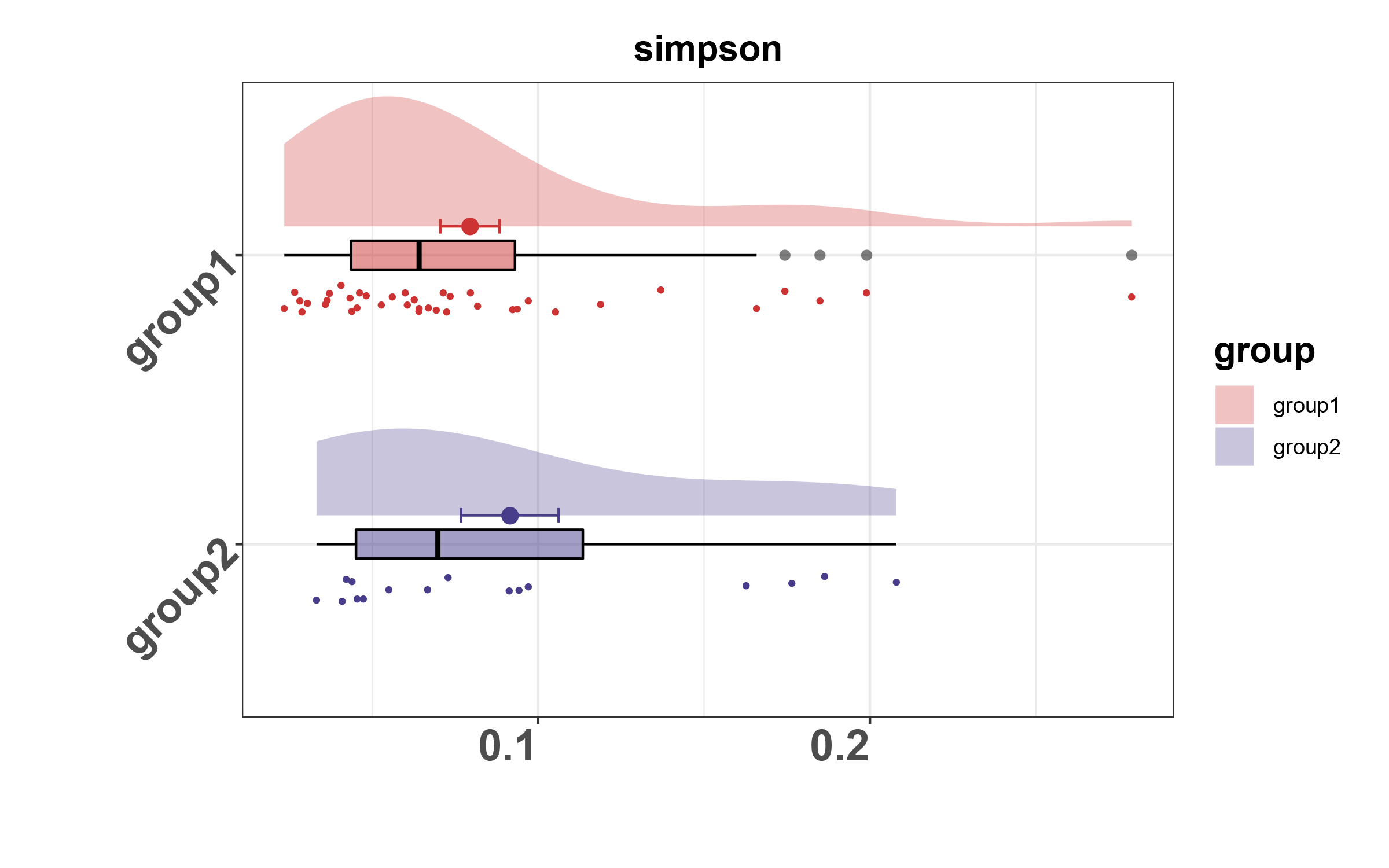

Supplement: Figures.zip [file IRNF_A_2514184_SM1332.zip › Figures/Supplemental Figure 1F.tif]

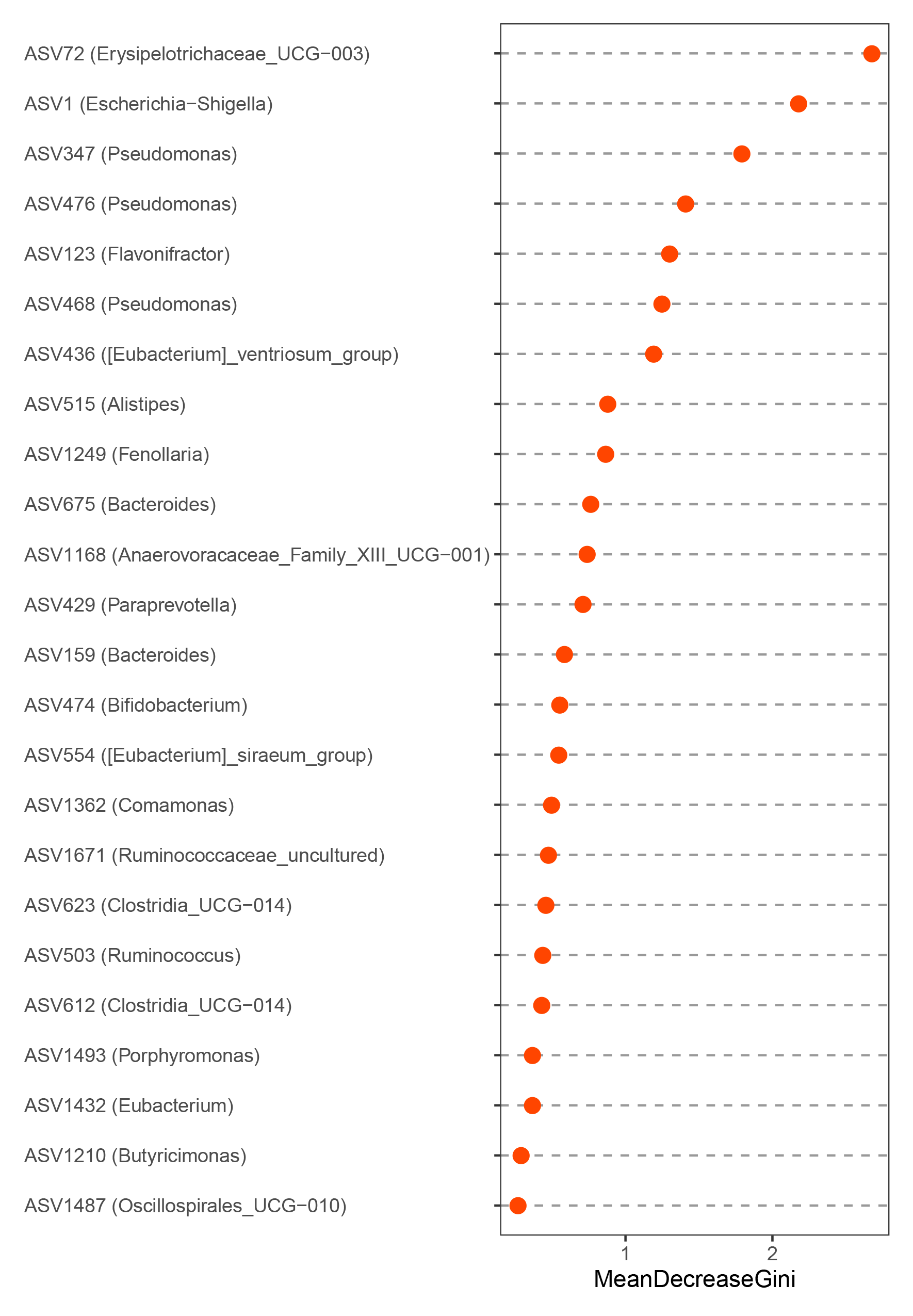

Supplement: Figures.zip [file IRNF_A_2514184_SM1332.zip › Figures/Supplemental Figure 2.tif]
